# BAG2 Inhibits Cervical Cancer Progression by Modulating Type I Interferon Signaling through Stabilizing STING

**DOI:** 10.1002/advs.202414637

**Published:** 2025-05-14

**Authors:** Shijie Yao, Siming Chen, Anjin Wang, Ziyan Liang, Xuelian Liu, Yang Gao, Hongbing Cai

**Affiliations:** ^1^ Department of Gynecological Oncology Zhongnan Hospital of Wuhan University Wuhan 430071 Hubei China; ^2^ Hubei Key Laboratory of Tumor Biological Behaviors Wuhan 430071 China; ^3^ Hubei Cancer Clinical Study Center Wuhan 430071 China; ^4^ Department of Urology Zhongnan Hospital of Wuhan University Wuhan 430071 China

**Keywords:** cervical cancer, STING, BAG2, STUB1, type I interferon response

## Abstract

Cervical cancer possesses high morbidity and mortality rates, and a comprehensive understanding of its molecular underpinnings is essential for advancing clinical management strategies. The innate immune sensor STING, which activates type I interferon signaling, plays a pivotal role in enhancing anti‐tumor activity. Despite increased attention to STING's involvement in cervical cancer, the regulatory mechanisms governing its protein homeostasis remain poorly understood. In this study, it is found that the BAG2‐STUB1 complex regulates ubiquitin proteasomal degradation of STING, which affects the development of cervical cancer. Mechanistically, BAG2 inhibits ubiquitination of STING and stabilizes it by interacting with STING. Specifically, BAG2 inhibits STUB1 from attaching the K48‐linked ubiquitin chains at K338 and K370 of STING by forming a complex with STUB1. Functionally, enhanced BAG2 expression suppresses cervical cancer progression by activating the type I interferon pathway in a STING‐dependent manner. Notably, clinical cervical cancer samples revealed a positive correlation between BAG2 and STING levels, with low BAG2 expression is strongly linked to advanced disease and poor prognosis in cervical cancer. Collectively, these findings elucidate the molecular mechanism by which the BAG2‐STUB1 complex regulates STING homeostasis, underscoring BAG2's potential as a diagnostic biomarker and therapeutic target in cervical cancer.

## Graphical abstract

Based on IP‐MS analysis, BAG2 is confirmed to be essential for ubiquitination and protein homeostasis regulation of STING in cervical cancer. BAG2 inhibits the ubiquitination and degradation of STING by forming a complex with STUB1, thereby activating the type I IFN signaling pathway and inhibiting the development of cervical cancer. Low BAG2 expression is associated with poor prognosis of cervical cancer.

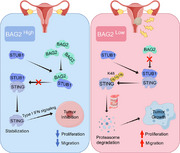

## 1 Introduction

Cervical cancer ranks as the fourth most prevalent malignant tumor among women worldwide, imposing a substantial burden on both public health and the economy.^[^ ^1^ ^]^ Infection with human papillomavirus (HPV) is the primary etiological factor for cervical cancer, particularly high‐risk HPV types, such as HPV16 and HPV18.^[^ ^2^ ^]^ The oncogenic effects of these viruses are largely mediated by the E6 and E7 oncoproteins.^[^ ^3^ ^]^ The HPV E6 protein accelerates the degradation of the tumor suppressor protein p53, thereby disrupting normal cell cycle regulation, inhibiting apoptosis, and promoting cancer cell proliferation.^[^ ^4^ ^]^ Meanwhile, the HPV E7 protein binds to the retinoblastoma protein (pRb), thereby releasing the E2F transcription factor from its inhibitory control, thereby driving uncontrolled cell cycle progression and further facilitating tumor growth.^[^ ^5^ ^]^ The clinical prognosis of cervical cancer is heavily dependent on early detection, with a five‐year survival rate exceeding 90% when diagnosed at an early stage. However, recurrence or distant metastasis significantly worsens the prognosis, leading to a marked reduction in survival rates.^[^ ^6^ ^]^ For early‐stage and locally invasive cases, radical hysterectomy combined with pelvic lymph node dissection, supplemented by chemotherapy and radiotherapy is the common standard treatment.^[^ ^7^ ^]^ In contrast, advanced cervical cancer presents a greater therapeutic challenge. Although systemic therapy and radiotherapy can extend survival, their efficacy remains limited. Patients with recurrent or metastatic disease often face a poor prognosis, with many only eligible for palliative care focused on symptom and pain management.^[^ ^8^ ^]^ A deeper understanding of the molecular mechanisms underlying cervical cancer is critical to uncovering its pathogenesis. Additionally, the identification of novel diagnostic and prognostic biomarkers could significantly improve cervical cancer early detection rates and provide a solid foundation for the development of personalized treatment approaches.

STING is a pivotal intracellular receptor that plays a significant role in the immune response.^[^ ^9^ ^]^ It detects abnormal DNA arising from DNA damage or viral infection, thereby activating TBK1,^[^ ^10^ ^]^ which subsequently phosphorylates IRF3.^[^ ^11^ ^]^ This cascade triggers type I interferons (e.g., IFN‐α/β)^[^ ^12^ ^]^ and initiates antiviral and anti‐tumor responses, including the recruitment of CD8^+^ T cells through chemokine release.^[^ ^13^ ^]^ The involvement of the STING pathway in cervical cancer has attracted considerable attention. Studies have revealed that activation of the STING‐TBK1 axis inhibits cervical cancer growth by promoting the ubiquitination‐mediated degradation of the E7 oncoprotein.^[^ ^14^ ^]^ Current research on the STING pathway predominantly focuses on its activation mechanisms and downstream immune responses.^[^ ^15^ ^]^ In prostate cancer, CDK4/6 impedes the STING signaling pathway by regulating TBK1 phosphorylation,^[^ ^16^ ^]^ while in breast cancer, ARIH1 activates STING‐mediated T‐cell responses, thereby enhancing tumor sensitivity to immune checkpoint blockade.^[^ ^17^ ^]^ The function of STING is regulated by various post‐translational modifications, such as ubiquitination and phosphorylation, which are essential for its structural integrity,^[^ ^18^ ^]^ functional activity,^[^ ^19^ ^]^ and immune response modulation.^[^ ^20^ ^]^ Although the molecular mechanisms of STING in various cancers are well‐documented, studies investigating the post‐translational modifications of STING in cervical cancer, particularly its protein homeostasis, remain scarce. Thus, this study aims to explore the key post‐translational modification partners of STING in cervical cancer and elucidate their regulatory mechanisms.

Bcl2‐associated athanogene 2 (BAG2), a member of the BAG family (comprising BAG1, BAG2, BAG3, BAG4, BAG5, and BAG6), is distinguished by its carboxy‐terminal BAG structural domain.^[^ ^21^ ^]^ As a multifunctional chaperone protein, BAG2 plays a key role in both cancer and degenerative diseases by regulating the stability and function of target proteins through direct interactions.^[^ ^22^ ^]^ Evidence indicates that BAG2 is a potent inhibitor of ubiquitin ligase STIP1 homology and U‐box‐containing protein 1 (STUB1). By binding to STUB1, BAG2 inhibits the binding of STUB1 to the substrate, thereby preventing substrate ubiquitin‐dependent degradation and maintaining the functional activity of the substrate.^[^ ^23^ ^]^ For instance, BAG2 has been shown to disrupt STUB1‐mediated ubiquitination and degradation of HSP72.^[^ ^24^ ^]^ Moreover, BAG2 reduces PINK1 degradation by inhibiting its ubiquitination, thereby stabilizing PINK1 protein level and promoting mitochondrial autophagy.^[^ ^25^ ^]^ In breast cancer, BAG2 contributes to chemoresistance by upregulating mutant p53 protein levels.^[^ ^26^ ^]^ Notably, high BAG2 expression has been shown to induce p21‐dependent cellular senescence and oncogenic arrest.^[^ ^27^ ^]^ While BAG2's role in stabilizing target proteins is well‐established, its specific function in cervical cancer, particularly in regulating STING protein homeostasis, remains to be further investigated.

## 2 Results

### 2.1 BAG2 is a Binding Partner of STING

**Figure 1:**
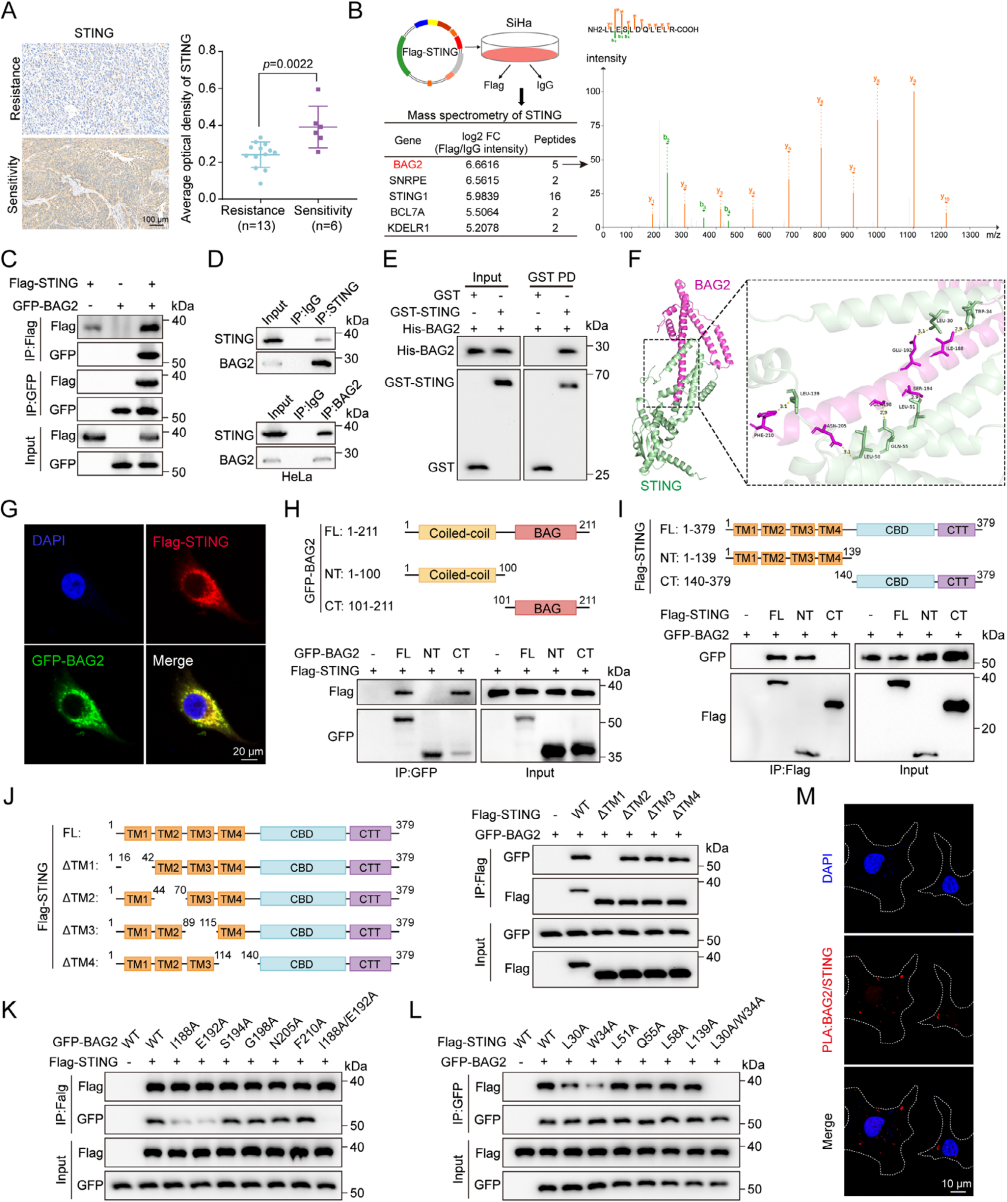
BAG2 is a binding partner for STING. A) Tissues from 19 cervical cancer patients who received immunotherapy and were assessed for sensitivity to treatment using the immune response evaluation criteria in solid tumors (iRECIST). STING IHC staining representative images (left panel) and statistical graphs (right panel) for cervical cancer immunotherapy‐sensitivity and immunotherapy‐resistance groups. Scale bar = 100 µm. STING average optical density (AOD) = integrated optical density (IOD)/positive staining area. B) After 48 hrs of Flag‐STING transfection, SiHa cells were lysed and the lysates were treated with IP‐Flag and IgG, respectively, and any interacting proteins were then identified by mass spectrometry. Schematic of IP‐MS for screening of interacting proteins with STING (left panel). Image of BAG2 from IP‐MS secondary ion mass spectrometry (right panel). C) HEK293T cells transfected with Flag‐STING and GFP‐BAG2 were incubated with MG132 (10 µm) for 6 h, and Western blot analysis of GFP‐BAG2 and Flag‐STING was performed after IP analysis with Flag and GFP antibodies. D) Western blot analysis of STING and BAG2 in HeLa cells after IP analysis with antibodies against STING (top panel) and BAG2 (bottom panel). E) Western blot analysis of His‐BAG2, GST‐STING after GST pull‐down assays. F) Protein‐protein docking of STING and BAG2. Red represents BAG2, green represents STING, and yellow represents hydrogen bonding. G) Fluorescence confocal imaging showing localization of GFP‐BAG2 (green) and Flag‐STING (red) in SiHa cells. Scale bar = 20 µm. H) Schematic representation of the GFP‐BAG2 truncations (top panel). HEK293T cells transfected with Flag‐STING and GFP‐BAG2 or its truncated mutants were incubated with MG132 (10 µm) for 6 h, and Western blot analysis of Flag‐STING was performed after IP analysis with GFP antibody (bottom panel). I) Schematic representation of the Flag‐STING truncations (top panel). HEK293T cells transfected with GFP‐BAG2 and Flag‐STING or its truncated mutants were incubated with MG132 (10 µm) for 6 h, and Western blot analysis of GFP‐BAG2 was performed after IP analysis with Flag antibody (bottom panel). **(J)** Schematic representation of the Flag‐STING deletion mutations (left panel). HEK293T cells transfected with GFP‐BAG2 and Flag‐STING or its deletion mutants were incubated with MG132 (10 µm) for 6 h, and Western blot analysis of GFP‐BAG2 was performed after IP analysis with Flag antibody (right panel). K) HEK293T cells transfected with Flag‐STING and GFP‐BAG2 or its point mutants were incubated with MG132 (10 µm) for 6 h, and Western blot analysis of GFP‐BAG2 was performed after IP analysis with Flag antibody. L) HEK293T cells transfected with GFP‐BAG2 and Flag‐STING or its point mutants were incubated with MG132 (10 µm) for 6 h, and Western blot analysis of Flag‐STING was performed after IP analysis with GFP antibody. M) PLA confocal microscopy images of STING and BAG2 interaction in HeLa cells. Scale bar = 10 µm. Statistical significance was determined by two‐tailed unpaired Student's t‐test (A).

STING, a key innate immune signaling factor, plays a crucial role in the antitumor immune response across various cancers.^[^ ^28^ ^]^ By comparing STING protein levels in cervical cancer tissues from immunotherapy‐sensitive and immunotherapy‐resistant patients, our study observed significantly higher STING protein levels in immunotherapy‐sensitive patients, suggesting a potential role for altered STING protein levels in clinical treatment response and prognostic evaluation in cervical cancer (**Figure** **1**A; and Table S1, Supporting Information). The functionality of STING protein is regulated by a variety of post‐translational modifications, which are critical for its stability, activity and immune response modulation.^[^ ^29^ ^]^ To explore the mechanisms underlying the post‐translational modification regulation of STING proteins in cervical cancer, immunoprecipitation‐mass spectrometry (IP‐MS) was employed to explore the crucial binding partners of STING proteins. Additionally, a Flag‐STING plasmid was constructed and transfected into SiHa cells, followed by affinity purification using an anti‐Flag antibody. To confirm the STING protein level, the immunoprecipitated samples were analyzed via silver staining, which clearly indicated successful detection of STING in the experimental group, while no STING signal was observed in the control group (Figure S1A, Supporting Information). Subsequently, IP‐MS analysis identified BAG2 as a prominent STING‐interacting protein with a high hit rate (Figure 1B). Previous research has demonstrated that BAG2 regulates the stability and function of target proteins by interacting with them. Specifically, BAG2 inhibits the ubiquitination of target proteins through its deubiquitination activity, thereby preventing their degradation and preserving their functional integrity.^[^ ^22^ ^]^ In view of this, we speculated whether BAG2 could act as a regulatory chaperone for STING, influencing its protein activity. Consequently, BAG2 was selected as a key candidate for further investigation.

To further confirm the interaction between BAG2 and STING, we co‐transfected Flag‐STING and GFP‐BAG2 plasmids into HEK293T cells and performed Co‐IP assay. Immunoblotting results revealed that BAG2 was successfully detected in the STING immunoprecipitate, and STING was similarly identified in the BAG2 immunoprecipitate (Figure 1C). Additionally, Co‐IP assays using anti‐BAG2 and anti‐STING antibodies in HeLa and SiHa cells further validated the reciprocal binding between BAG2 and STING (Figure 1D; Figure S1B, Supporting Information). These results strongly support the existence of an interaction between BAG2 and STING. Notably, a GST pull‐down assay demonstrated direct binding between recombinant GST‐STING and recombinant His‐BAG2 in vitro (Figure 1E). Protein molecular docking simulations revealed that the C‐terminal BAG structural domain of BAG2 primarily interacts with the N‐terminal transmembrane structural domain of STING. Detailed structural interaction analysis indicated that residues L30, W34, L51, Q55, L58, and L139 of STING closely interacted with residues E192, I188, S194, G198, N205, and F210 of BAG2 through multiple hydrogen bonds (Figure 1F). Specific docking interaction sites are detailed in Table S2 (Supporting Information). Immunofluorescence co‐localization analysis showed that STING and BAG2 co‐localized in the cytoplasm (Figure 1G). To pinpoint the precise structural domains interacting of STING and BAG2, truncations of both STING and BAG2 were constructed for Co‐IP analysis. The transmembrane domain of STING (amino acids 1–139) and the BAG domain of BAG2 (amino acids 101–211) were found to be essential for their association (Figure 1H,I). Interestingly, the TM1 region (amino acids 17–41) within the transmembrane domain of STING was critical for binding to BAG2 (Figure 1J). To further investigate the specific binding sites of BAG2 and STING, point mutations were introduced in both proteins based on the predicted interaction sites from the molecular docking results. Co‐IP assays demonstrated that mutating amino acid residues I188 and E192 in BAG2 alone significantly weakened its binding to STING, an interaction that was almost completely eliminated when both residues were mutated simultaneously (Figure 1K). Similarly, mutation of amino acid residues L30 and W34 in STING alone weakened its binding to BAG2, and the interaction was almost completely abolished when both amino acid residues were mutated simultaneously (Figure 1L). These results indicate that residues I188 and E192 in BAG2 and residues W34 and L30 in STING are critical for their interaction. Furthermore, the proximity ligation assay (PLA) confirmed a direct in situ interaction between BAG2 and STING (Figure 1M). Altogether, these results demonstrate a direct interaction between BAG2 and STING.

### 2.2 BAG2 Maintains STING Stability through Deubiquitination

**Figure 2:**
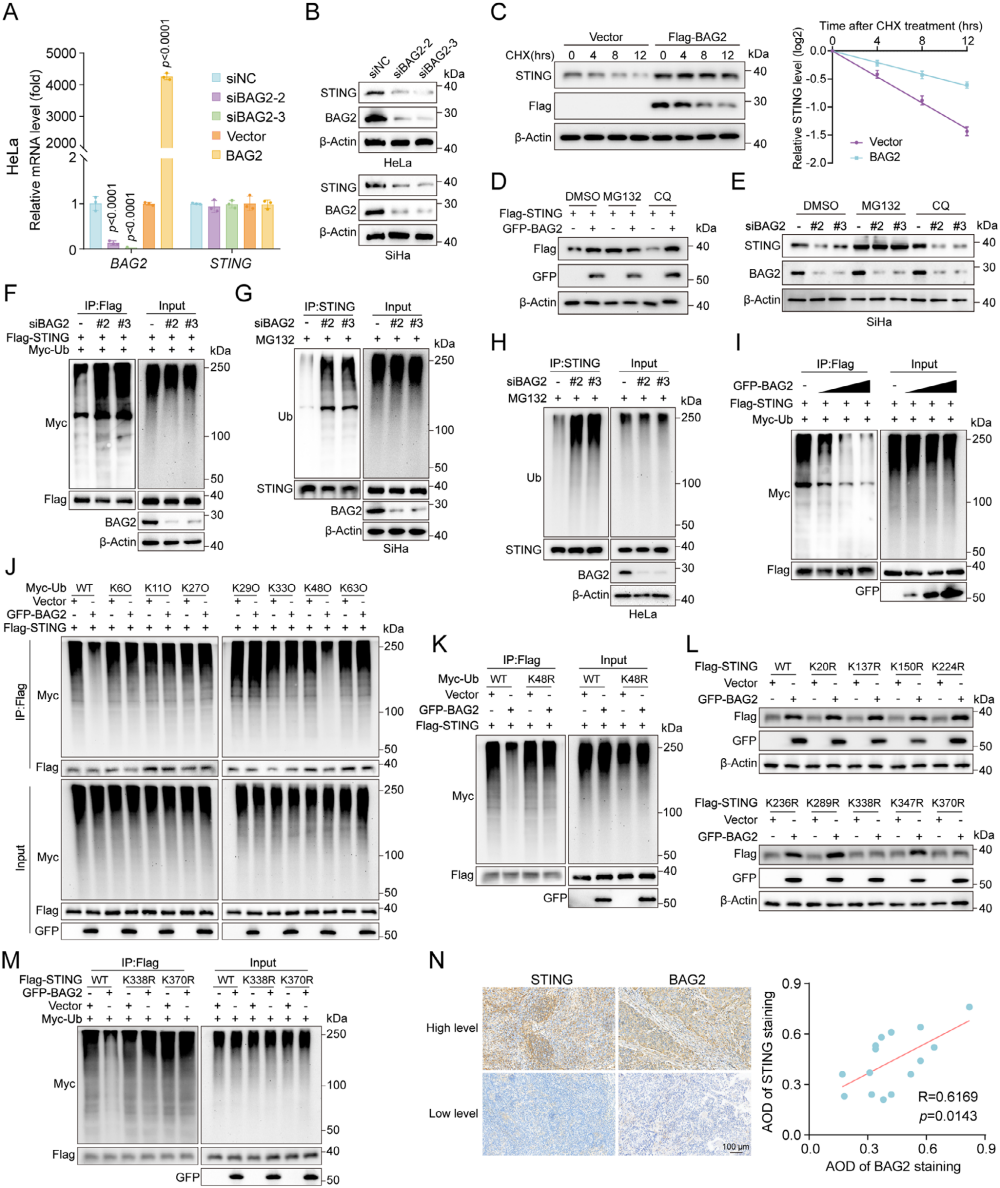
BAG2 inhibits ubiquitination and degradation of STING. A) Following BAG2 knockdown or overexpression, qRT‐PCR was utilized to measure the mRNA expression levels of *STING* and *BAG2* in HeLa cells. B) After transfection of HeLa and SiHa cells with *siBAG2*, STING protein levels were detected by Western blot. C) 50 µg mL^−1^ cycloheximide (CHX) was used to treat HEK293T cells transfected with Flag‐BAG2, and the cells were taken out at the designated intervals. Representative Western blot plots showing the impact of BAG2 overexpression on STING degradation (left panel) and protein half‐life statistics (right panel). D) HEK293T cells transfected with Flag‐STING and Vector/GFP‐BAG2 were exposed to DMSO, MG132 (10 µm), or chloroquine (CQ, 10 µm) for 6 h, after which Flag‐STING protein level was analyzed by Western blot. E) SiHa cells transfected with *siBAG2* were exposed to DMSO, MG132 (10 µm), or chloroquine (CQ, 10 µm) for 6 h, after which STING protein level was analyzed by Western blot. F) Flag‐STING, Myc‐ubiquitin (Myc‐Ub) and *siBAG2* (*siBAG2‐2* or *siBAG2‐3*) were transfected into HEK293T cells for 48 hrs, and MG132 was used to pretreat the cells for 6 h. The cell lysate was subjected to Co‐IP using anti‐Flag antibody and then Western blot assay using anti‐Myc antibody. G) SiHa cells transfected with *siBAG2* were exposed to MG132 (10 µm) for 6 h. The cell lysate was subjected to Co‐IP using anti‐STING antibody and then Western blot assay using anti‐Ub antibody. H) HeLa cells transfected with *siBAG2* were exposed to MG132 (10 µm) for 6 h. The cell lysate was subjected to Co‐IP using anti‐STING antibody and then Western blot assay using anti‐Ub antibody. I) HEK293T cells transfected with GFP‐BAG2 (0.2, 1.0, or 4.0 µg), Flag‐STING and Myc‐Ub were exposed to MG132 (10 µm) for 6 h. The cell lysate was subjected to Co‐IP using anti‐Flag antibody and then Western blot assay using anti‐Myc antibody. J) HEK293T cells transfected with Flag‐STING, Vector or GFP‐BAG2, Myc‐Ub‐WT or Myc‐Ub‐Mut (K6O or K11O or K27O or K29O or K33O or K48O or K63O) were exposed to MG132 (10 µm) for 6 h. The cell lysate was subjected to Co‐IP using anti‐Flag antibody and then Western blot assay using anti‐Myc antibody. K) HEK293T cells transfected with Flag‐STING, Vector or GFP‐BAG2, Myc‐Ub‐WT or Myc‐Ub‐K48R were exposed to MG132 (10 µm) for 6 h. The cell lysate was subjected to Co‐IP using anti‐Flag antibody and then Western blot assay using anti‐Myc antibody. L) HEK293T cells were transfected with Flag‐STING or its mutations and Vector or GFP‐BAG2, and Western blot analysis of Flag‐STING was performed. M) HEK293T cells transfected with Flag‐STING or its mutations, Vector or GFP‐BAG2 and Myc‐Ub were exposed to MG132 (10 µm) for 6 h. The cell lysate was subjected to Co‐IP using anti‐Flag antibody and then Western blot assay using anti‐Myc antibody. N) Representative images (left panel) and statistical graph (right panel) showing IHC staining for BAG2 and STING in cervical cancer tissues. Scale bar = 100 µm. Pearson test was used for correlation analysis. Data are shown as mean ± SD, with *n* = 3 (A, C) biological independent experiments. The one‐way ANOVA with Dunnett's multiple comparisons test was utilized to ascertain statistical differences within the *BAG2* knockdown group, the two‐tailed unpaired Student's t‐test was used to assess the statistical difference for the BAG2 overexpression group (A).

Given BAG2's role as a regulator of chaperone function, the mechanism by which BAG2 modulates STING was investigated. To validate the regulatory effect of BAG2 on STING, three siRNAs targeting BAG2 (*siBAG2‐1, siBAG2‐2*, and *siBAG2‐3*) were designed, and a BAG2 overexpression plasmid was constructed. We then employed qRT‐PCR and Western blot to assess the efficiency of BAG2 knockdown or overexpression. Based on these results, the two most effective siRNAs (*siBAG2‐2* and *siBAG2‐3*) were selected for further experiments (Figure S1C–G, Supporting Information). Notably, the overexpression and knockdown of BAG2 significantly altered the STING protein levels in both SiHa and HeLa cells, with no effect on *STING* mRNA expression (**Figure** **2**A,B; Figure S1H,I, Supporting Information). And overexpression of BAG2 led to a dose‐dependent increase in STING protein levels (Figure S1J, Supporting Information). Moreover, we employed cycloheximide (CHX) to inhibit protein synthesis and examined the impact of BAG2 on STING protein stability. The results revealed that overexpression of BAG2 significantly extended the half‐life of STING (Figure 2C), while knockdown of *BAG2* shortened its half‐life (Figure S1K,L, Supporting Information), suggesting that BAG2 stabilizes STING through a specific mechanism. Furthermore, the proteasome inhibitor (MG132) effectively blocked the upregulation of STING induced by BAG2 overexpression (Figure 2D) and inhibited the downregulation of STING resulting from *BAG2* knockdown (Figure 2E). In contrast, the autophagolysosomal inhibitor chloroquine (CQ) did not affect STING stability. These results suggest that BAG2 inhibits STING protein degradation through the ubiquitin‐proteasome pathway.

Subsequent experiments explored the influence of BAG2 on STING ubiquitination. Ubiquitination assays demonstrated that *BAG2* knockdown markedly elevated STING ubiquitination in HEK293T cells (Figure 2F). Similarly, in SiHa and HeLa cells, *BAG2* knockdown also increased the ubiquitination of endogenous STING (Figure 2G,H). In contrast, BAG2 overexpression resulted in a substantial reduction of STING ubiquitination, with the extent of the decrease being dose‐dependent (Figure 2I). Given the distinct functional roles of different ubiquitin chain types, further investigations were conducted to determine which types of ubiquitin chains participated in BAG2‐mediated STING deubiquitination. Expression plasmids for ubiquitin mutants retaining only a single lysine residue, including K6O, K11O, K27O, K29O, K33O, K48O, and K63O, were transfected into cells together with the BAG2 plasmid. The findings indicated that BAG2 predominantly facilitated the deubiquitination of STING through the K48O mutant (in which only the Lys residue 48 was retained), implying that BAG2 predominantly removes K48‐linked ubiquitin chains from STING (Figure 2J). This finding was further corroborated by the K48R mutant (in which only Lys residue 48 was mutated to Arg), in which the K48R mutant blocked BAG2‐induced deubiquitination of the STING (Figure 2K). To pinpoint specific ubiquitination sites on STING, each lysine residue was substituted with arginine and transfected these mutants into HEK293T cells for subsequent analysis. The results showed that BAG2 promoted the expression of WT‐STING as well as all single lysine mutants (K20R, K137R, K150R, K224R, K236R, K289R, and K347R), except for the K338R and K370R mutants (Figure 2L). Notably, BAG2 was ineffective in inhibiting STING ubiquitination in the K338R and K370R mutants (Figure 2M), suggesting that these mutants are resistant to BAG2‐mediated deubiquitination and that BAG2 primarily targets the K338 and K370 sites on STING. Notably, the protein levels of BAG2 and STING were quantitatively assessed by IHC in cervical cancer tissue samples from the Zhongnan Hospital cohort, and a strong positive correlation was found between the protein levels of BAG2 and STING in cervical cancer tissues (Figure 2N; and Table S3, Supporting Information). In summary, BAG2 stabilizes STING by removing K48‐linked ubiquitin chains from the K338 and K370 sites of STING.

### 2.3 BAG2 Inhibits STUB1‐Mediated STING Ubiquitination and Degradation

**Figure 3:**
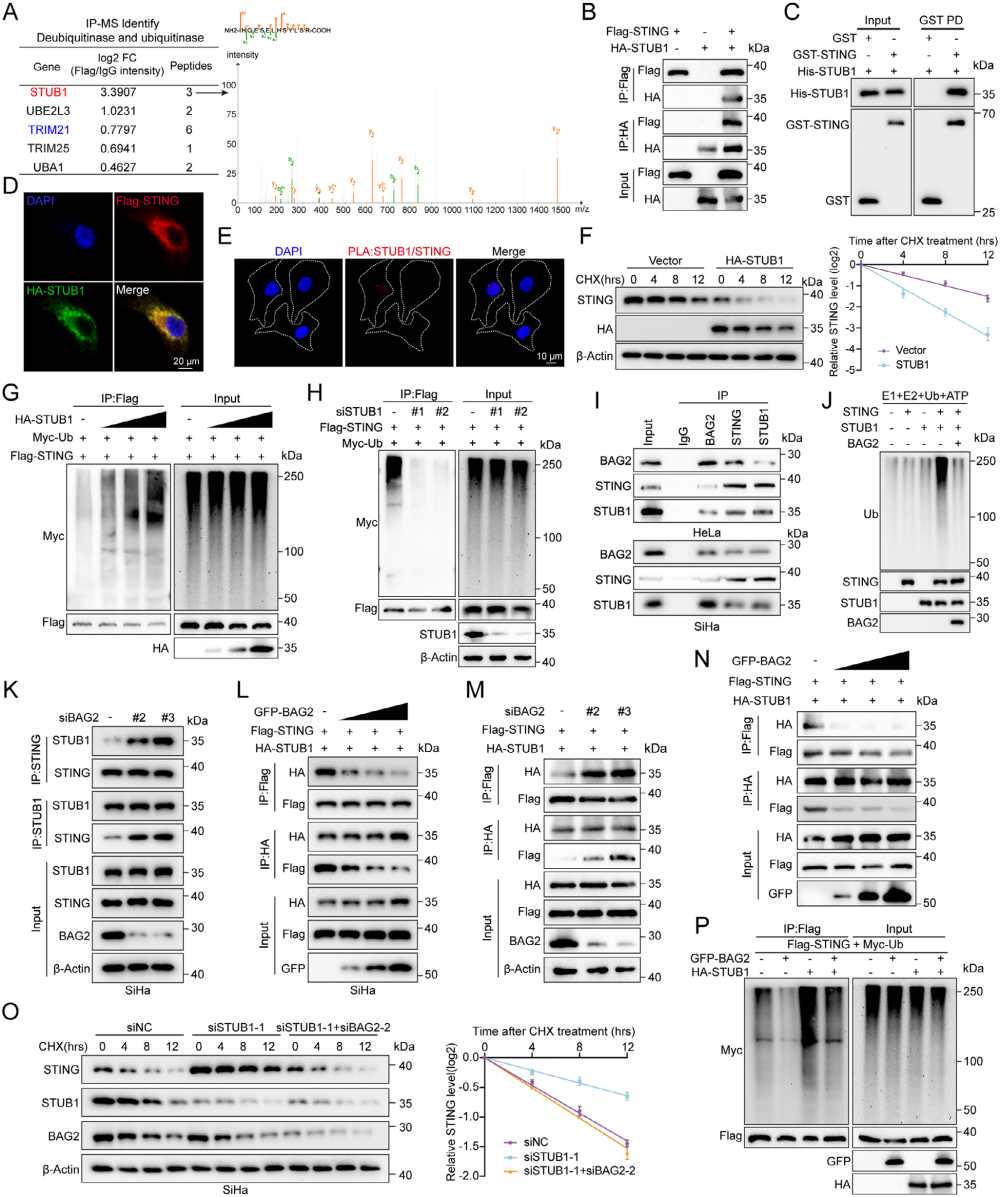
BAG2 inhibits STUB1‐mediated ubiquitination and degradation of STING. A) IP‐MS assay to screen for ubiquitinases or deubiquitinases interacting with STING (left panel). Image of STUB1 from IP‐MS secondary ion mass spectrometry (right panel). B) HEK293T cells transfected with Flag‐STING and HA‐STUB1 were incubated with MG132 (10 µm) for 6 h, and Western blot analysis of HA‐STUB1 and Flag‐STING was performed after IP analysis with Flag and HA antibodies. C) Western blot analysis of His‐STUB1, GST‐STING after GST pull‐down assays. D) Fluorescence confocal imaging showing localization of HA‐STUB1 (green) and Flag‐STING (red) in SiHa cells. Scale bar = 20 µm. E) PLA confocal microscopy images of STING and STUB1 interaction in HeLa cells. Scale bar = 10 µm. F) 50 µg mL^−1^ CHX was used to treat HEK293T cells transfected with HA‐STUB1, and the cells were taken out at the designated intervals. Representative Western blot plots showing the impact of STUB1 overexpression on STING degradation (left panel) and protein half‐life statistics (right panel). G) HEK293T cells transfected with HA‐STUB1 (0.2, 1.0, or 4.0 µg), Flag‐STING and Myc‐Ub were exposed to MG132 (10 µm) for 6 h. The cell lysate was subjected to Co‐IP using anti‐Flag antibody and then Western blot assay using anti‐Myc antibody. H) Flag‐STING, Myc‐Ub and *siSTUB1* were transfected into HEK293T cells for 48 h, and MG132 was used to pretreat the cells for 6 h. The cell lysate was subjected to Co‐IP using anti‐Flag antibody and then Western blot assay using anti‐Myc antibody. I) Western blot analysis was used to determine the protein levels of BAG2, STING, and STUB1 in SiHa and HeLa cells treated with IgG‐IP, BAG2‐IP, STING‐IP, or STUB1‐IP. J) In vitro ubiquitination of STING was measured through incubating STING protein with E1, E2, and biotinylated‐Ub combined with or without STUB1 protein and BAG2 protein, and subsequent Western blot analyses. K) SiHa cells transfected with *siBAG2* were incubated with MG132 (10 µm) for 6 h, and Western blot analysis of STUB1 and STING was performed after IP analysis with STING and STUB1 antibodies. L) SiHa cells transfected with Flag‐STING, HA‐STUB1 and GFP‐BAG2 (0.2, 1.0, or 4.0 µg) were incubated with MG132 (10 µm) for 6 h, and Western blot analysis of HA‐STUB1 and Flag‐STING was performed after IP analysis with Flag and HA antibodies. M) HEK293T cells transfected with Flag‐STING, HA‐STUB1 and *siBAG2* were incubated with MG132 (10 µm) for 6 h, and Western blot analysis of HA‐STUB1 and Flag‐STING was performed after IP analysis with Flag and HA antibodies. N) HEK293T cells transfected with Flag‐STING, HA‐STUB1 and GFP‐BAG2 (0.2, 1.0, or 4.0 µg) were incubated with MG132 (10 µm) for 6 h, and Western blot analysis of HA‐STUB1 and Flag‐STING was performed after IP analysis with Flag and HA antibodies. O) SiHa cells transfected with *siSTUB1‐1*, *siSTUB1‐1+siBAG2‐2* were incubated with CHX (50 µg mL^−1^) for indicated times. Representative Western blot plots showing the combined effect of STUB1 and BAG2 on STING degradation (left panel) and protein half‐life statistics (right panel). P) HEK293T cells transfected with Flag‐STING, Myc‐Ub, HA‐STUB1 or/and GFP‐BAG2 were exposed to MG132 (10 µm) for 6 h. The cell lysate was subjected to Co‐IP using anti‐Flag antibody and then Western blot assay using anti‐Myc antibody. Data are shown as mean ± SD, with *n* = 3 (F, O) biological independent experiments.

Our findings suggest that BAG2 binds to STING and cleaves K48‐linked ubiquitin chains to regulate its stability. Since BAG2 has not been shown to directly regulate protein stability in terms of molecular function, we hypothesized that BAG2 regulates protein stability in STING through specific ubiquitinases or deubiquitinases. To test this hypothesis, we screened for ubiquitinases or deubiquitinases interacting with STING from IP‐MS assays. The results indicated that STUB1 is the E3 ubiquitin ligase that is most likely to attach to STING. Notably, the previously reported ubiquitin E3 ligase of STING‐interacting proteins, TRIM21,^[^ ^30^ ^]^ was identified, confirming the validity of our screen for STING‐interacting proteins (**Figure** **3**A). Interestingly, it has been reported that BAG2 can form a complex with STUB1 and inhibit ubiquitination and degradation of downstream target proteins by STUB1.^[^ ^24^ ^]^ Therefore, STUB1 was chosen as a potential molecule for further study. As we expected, a strong binding affinity between STUB1 and STING was confirmed in HEK293T cells by exogenous Co‐IP assay (Figure 3B). In addition, endogenous interactions between STUB1 and STING were confirmed in SiHa and HeLa cells (Figure S2A, Supporting Information). Importantly, GST pull‐down assay showed direct binding of recombinant GST‐STING and recombinant His‐STUB1 in vitro (Figure 3C). Immunofluorescence co‐localization analysis showed that STUB1 and STING also co‐localized in the cytoplasm (Figure 3D). PLA assay also confirmed the direct interaction between STUB1 and STING (Figure 3E). These results indicate that STUB1 directly binds to STING.

Next, we investigated whether STUB1 plays a role in the degradation of STING. We constructed and validated two siRNAs targeting STUB1 (*siSTUB1‐1* and *siSTUB1‐2*) and STUB1 overexpression plasmid (Figure S2B, Supporting Information). Notably, the knockdown or overexpression of STUB1 did not significantly alter *STING* mRNA expression, but it significantly affected STING protein levels (Figure S2B,C, Supporting Information). Importantly, the overexpression of STUB1 resulted in a dose‐dependent decrease in STING protein levels (Figure S2D, Supporting Information). The CHX‐chase assay also confirmed that STUB1 overexpression significantly shortened the half‐life of STING (Figure 3F), and conversely, knockdown of *STUB1* significantly lengthened the half‐life of STING (Figure S2E, Supporting Information), indicating that STUB1 regulates STING protein stability. The effect of STUB1 on STING ubiquitination was also investigated. Remarkably, overexpression of STUB1 led to an increase in STING ubiquitination levels, and this increase was concentration gradient dependent (Figure 3G). Conversely, knockdown of *STUB1* significantly reduced the ubiquitination level of STING (Figure 3H). These results suggest that STUB1 promotes the ubiquitination of STING, thereby facilitating its degradation and increasing its instability.

Next, we further explored whether BAG2 could inhibit STUB1‐mediated STING ubiquitination and degradation. We verified the connection between BAG2, STUB1, and STING in SiHa and HeLa cells by endogenous Co‐IP assays (Figure 3I). To provide direct evidence of STUB1‐mediated STING ubiquitination, STING proteins were co‐incubated with E1, E2, and biotinylated‐Ub, and subjected to an in vitro ubiquitination assay, both with and without STUB1. This analysis revealed that STUB1 directly induces STING ubiquitination in vitro, while BAG2 significantly counteracted this effect (Figure 3J). These results prompted the hypothesis that BAG2 inhibits STUB1‐mediated STING ubiquitination and degradation by disrupting the STUB1‐STING interaction. Consistent with this hypothesis, *BAG2* knockdown significantly increased STUB1 binding to STING in SiHa and HeLa cells (Figure 3K; Figure S2F, Supporting Information), whereas overexpression of BAG2 resulted in a concentration‐dependent decrease in STUB1 binding to STING (Figure 3L; Figure S2G, Supporting Information). Similar results were confirmed in HEK293T cells (Figure 3M,N). Additionally, in SiHa cells with *STUB1* knockdown, *BAG2* knockdown significantly restored the increased abundance and stability of STING proteins caused by *STUB1* depletion (Figure 3O). Notably, in vivo ubiquitination assay also showed that upregulation of BAG2 significantly attenuated the effect of STUB1 on STING ubiquitination (Figure 3P). These findings suggest that BAG2 stabilizes STING by inhibiting STUB1‐mediated STING ubiquitination, a process that is dependent on the presence of STUB1.

### 2.4 BAG2 is Associated with Progression and Prognosis in Cervical Cancer

**Figure 4:**
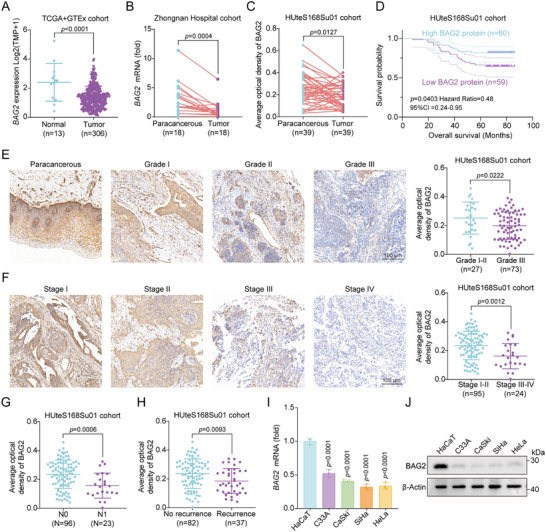
BAG2 is associated with prognosis in cervical cancer. A) *BAG2* mRNA expression in TCGA‐CESC cohort normal cervical tissues and cervical cancer. TPM: transcripts per million kilobases. B) *BAG2* mRNA expression in paracancerous cervical tissues (*n* = 18) and cervical cancer (*n* = 18) in the Zhongnan Hospital cohort (qRT‐PCR data). C) HUteS168Su01 tissue microarrays were used for the IHC identification of paired paracancerous cervical tissues (*n* = 39) and cervical cancer tissues (*n* = 39). D) An analysis was conducted on the overall survival of patients with cervical cancer in the HUteS168Su01 cohort across various BAG2 protein level groups. Based on the median BAG2 protein levels, patients were divided into BAG2 high group and BAG2 low group. The group exhibiting high BAG2 expression is shown by the blue line, whereas the group exhibiting low BAG2 expression is represented by the purple line. E) Representative images (left panel) and statistical graph (right panel) of IHC staining analysis of BAG2 protein levels in the HUteS168Su01 cohort with different pathological grades (Paracancerous, Grades I, II, and III). F) Representative images (left panel) and statistical graph (right panel) of IHC staining analysis of BAG2 protein levels at different stages of the HUteS168Su01 cohort (7th edition of the AJCC: Stage I, II, III, and IV). G,H) Statistical plots of IHC analyses of BAG2 expression levels in the HUteS168Su01 cohort grouped by **(G)** N stage (N0 and N1) and **(H)** recurrence status (No recurrence and Recurrence). I) *BAG2* mRNA expression in human non‐cancerous keratinocyte cell line (HaCaT) and cervical cancer cell lines (C33A, CaSki, SiHa and HeLa) were detected using qRT‐PCR. J) BAG2 protein level in HaCaT cell line and cervical cancer cell lines were detected using Western blot analysis. BAG2 average optical density (AOD) = integrated optical density (IOD)/area of positive staining (C‐H). Data are shown as mean ± SD, with *n* = 3 (I) biological independent experiments. Statistical significance was determined by two‐tailed unpaired Student's t‐test (A, E‐H), two‐tailed paired Student's t‐test (B‐C), one‐way ANOVA with Dunnett's multiple comparisons test (I), or the log‐rank test of Kaplan–Meier analysis (D).

Given the significance of BAG2 in the regulation of STING, we decided to investigate the relationship between BAG2 and major clinical characteristics of cervical cancer. The TIMER2 database showed that BAG2 expression is down‐regulated in most tumor types, especially in genitourinary tumors (including BLCA, CESC, KICH, KIRC, PRAD and UCEC) (Figure S3A, Supporting Information). Notably, BAG2 expression was significantly reduced in cervical cancer, while no significant changes were observed in other BAG family members (BAG1, BAG3, BAG4, BAG5, and BAG6), suggesting a unique role for BAG2 in cervical cancer (Figure S3B, Supporting Information). We collected extensive data to investigate the relationship between BAG2 and clinical parameters in cervical cancer. In the TCGA‐GTEx (normal: *n* = 13, tumor: *n* = 306) and Zhongnan hospital cohorts (paracancerous: *n* = 18, tumor: *n* = 18), *BAG2* mRNA expression levels were usually down‐regulated in cervical cancer tissues (**Figure** **4**A,B; and Table S4, Supporting Information). In addition, IHC analysis of paired tumor tissues from the HUteS168Su01 cohort (paracancerous: *n* = 39, tumor: *n* = 39) showed that the protein level of BAG2 was significantly down‐regulated in tumor tissues (Figure 4C; and Table S5, Supporting Information). In terms of prognosis assessment, we investigated the predictive value of BAG2 in cervical cancer prognosis by Kaplan‐Meier survival analysis. The results showed a significant positive association between BAG2 protein levels and overall cervical cancer survival in the HUteS168Su01 cohort (HR = 0.48, 95% CI = 0.24‐0.95, *p* = 0.0403) (Figure 4D). We further investigated the association between BAG2 protein levels and clinicopathological features of cervical cancer. The results showed that BAG2 protein level was negatively correlated with clinical characteristics of cervical cancer, including Grade, Stage and N stage (Figure 4E–G). Importantly, the tumor recurrence group's BAG2 protein levels were lower than those of the no recurrence group (Figure 4H). In addition, the UALCAN database showed that the mRNA expression of *BAG2* was lower in N1 stage compared to N0 stage (Figure S3C, Supporting Information). In addition, it is worth noting that methylation levels in the BAG2 promoter region were significantly elevated in cervical cancer tissues (Figure S3D, Supporting Information), suggesting a potential epigenetic regulation of BAG2 during cervical cancer progression. The mRNA and protein levels of BAG2 were evaluated in different cervical cancer cell lines (including C33A, CaSki, HeLa, and SiHa) and human non‐cancerous keratinocyte cell line (HaCaT). The results indicated that BAG2 mRNA expression and protein levels were significantly lower in cervical cancer cell lines compared to HaCaT cell line (Figure 4I,J). In conclusion, the data strongly suggest a correlation between BAG2 and cervical cancer progression and prognosis, highlighting that diminished BAG2 expression may contribute to the malignant progression and poor prognosis of the disease.

### 2.5 BAG2 Prevents the Proliferation and Metastasis of Cervical Cancer both In Vivo and In Vitro

**Figure 5:**
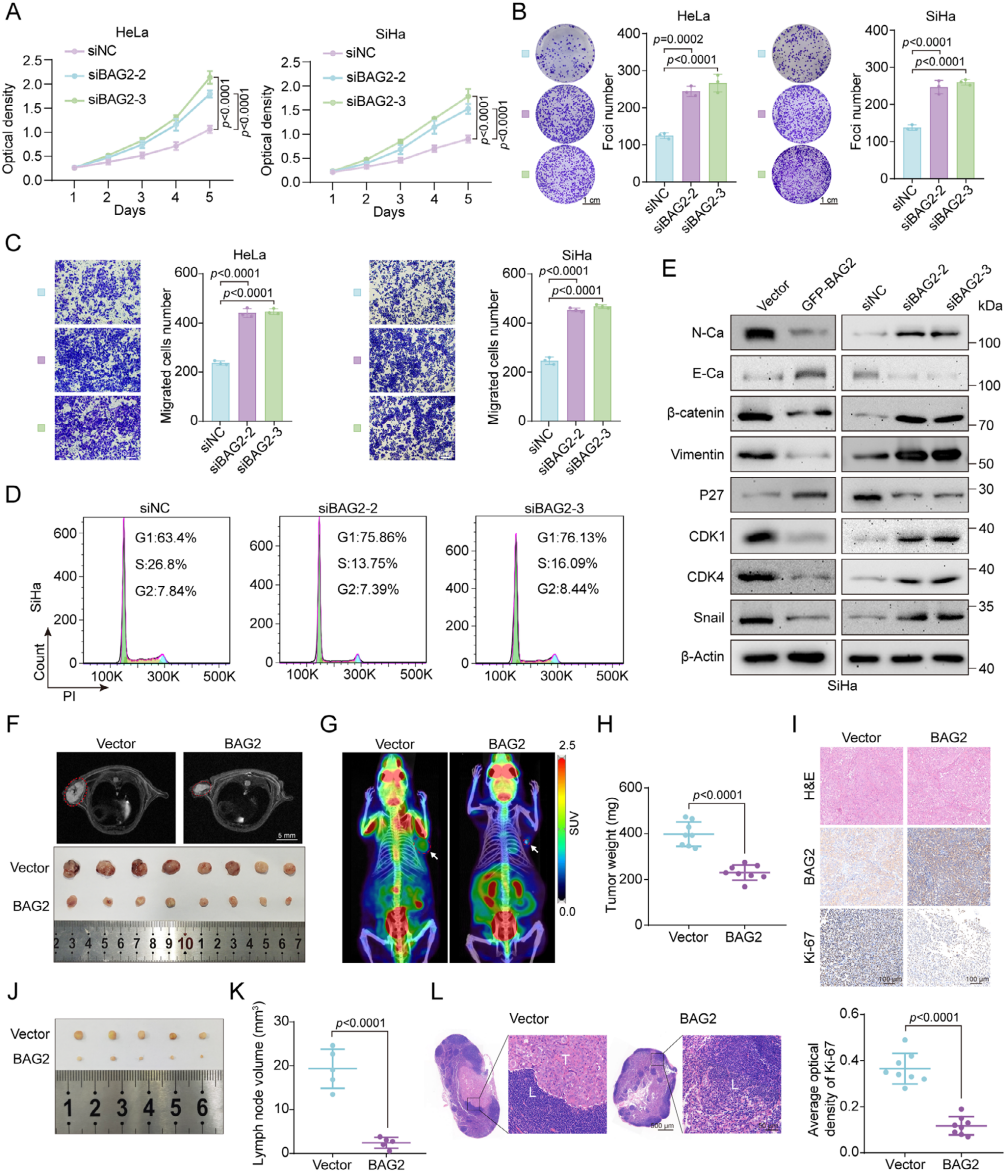
BAG2 prevents the proliferation and metastasis of cervical cancer both in vivo and in vitro. A) The proliferation capacity of SiHa and HeLa cells after *BAG2* knockdown was determined by CCK8 assay. B) Foci formation assay of proliferative capacity of SiHa and HeLa cells after *BAG2* knockdown treatment (left panel), followed by statistical analysis (right panel). C) Transwell assay showing migratory capacity of SiHa and HeLa cells after *BAG2* knockdown (left panel), followed by statistical analysis (right panel). D) Flow cytometry analysis of the effect of *BAG2* knockdown on SiHa cells cycle progression. E) Western blot analysis of proteins associated with cell cycle and EMT following BAG2 overexpression or knockdown in SiHa cells. F) Magnetic resonance imaging (MRI) images (top panel) and tissue anatomy images (bottom panel) showing subcutaneous graft tumors in the control and BAG2 groups. G) ^18^F‐FDG PET/CT scans were used to assess tumor growth and malignancy in each group of mice. H) Weight of subcutaneous tumors affected by overexpression of BAG2. I) H&E and IHC staining analysis of subcutaneous tumors (top panel). Statistical plot of Ki‐67 IHC staining in subcutaneous tumor tissue (bottom panel). J) Anatomical images of different groups of lymph nodes in the popliteal lymph node metastasis model constructed using the BAG2 overexpression stably transfected U14 cell line. K) Effect of BAG2 overexpression on lymph node metastatic volume. L) Analysis of mice popliteal lymph nodes stained with H&E. T = metastasized tumor tissue, L = normal lymph node tissue. Data are presented as mean values ± SD, with *n* = 3 (B‐D), 5 (K), 6 (A), or 8 (H, I) biological independent experiments. Statistical significance was determined by two‐tailed unpaired Student's t‐test (H, I, K), or one‐way ANOVA with Dunnett's multiple comparisons test (A‐C).

In order to carry out additional research on the function of BAG2 in controlling the phenotype of cervical cancer, BAG2 was knocked down and overexpressed in cervical cancer cell lines. According to CCK8 assays, BAG2 overexpression and knockdown respectively markedly reduced and increased the proliferation of SiHa and HeLa cells (**Figure** **5**A; Figure S4A, Supporting Information). The same results were obtained in foci formation assay (Figure 5B; Figure S4B, Supporting Information). Furthermore, transwell assays demonstrated a notable improvement in the migratory capacity of HeLa and SiHa cells following *BAG2* knockdown, in contrast to the considerable decrease in migratory capacity caused by BAG2 overexpression (Figure 5C; Figure S4C, Supporting Information). Flow cytometry results showed that knockdown of *BAG2* in SiHa cells led to an increase in the proportion of G1‐phase cells, but a decrease in G1‐phase cells when BAG2 was overexpressed (Figure 5D; Figure S4D,E, Supporting Information). Western blot was then employed to identify the level of proteins linked to the cell cycle and EMT. Knockdown of *BAG2* in SiHa and HeLa cells resulted in a significant increase in N‐cadherin, β‐catenin, Vimentin, CDK1, CDK4, and Snail protein levels, while E‐cadherin and P27 levels decreased. Conversely, BAG2 overexpression produced the opposite effects (Figure 5E; Figure S4F, Supporting Information).

To investigate the role of BAG2 in cervical cancer proliferation and metastasis in vivo, a subcutaneous tumor model and a popliteal lymph node metastasis model were established. Stable cell lines overexpressing BAG2 and Vector were generated in U14 cells, with the overexpression efficiency confirmed prior to further experiments (Figure S4G, Supporting Information). Magnetic resonance imaging (MRI) and anatomical tissue results revealed that BAG2 overexpression significantly reduced subcutaneous tumor weight and volume compared to controls (Figure 5F–H; Figure S4H, Supporting Information). Notably, PET/CT scans demonstrated significantly less tumor activity and malignancy in mice in the BAG2 overexpression group compared to the control group (Figure 5G). IHC staining of tumor tissues revealed reduced Ki67 protein levels in the BAG2 overexpression group (Figure 5I). Notably, we injected Vector and BAG2 stable cells into the footpads of mice to construct a popliteal lymph node metastasis model, significantly smaller popliteal lymph nodes were observed in the BAG2 overexpression group compared to controls (Figure 5J,K; Figure S4I, Supporting Information). H&E staining provided additional evidence that the BAG2 group had fewer tumor lesions than the Vector group (Figure 5L). These results collectively support the conclusion that BAG2 inhibits the proliferation and metastasis of cervical cancer cells.

### 2.6 BAG2 Positively Regulates STING‐Mediated Type I Interferon Responses

**Figure 6:**
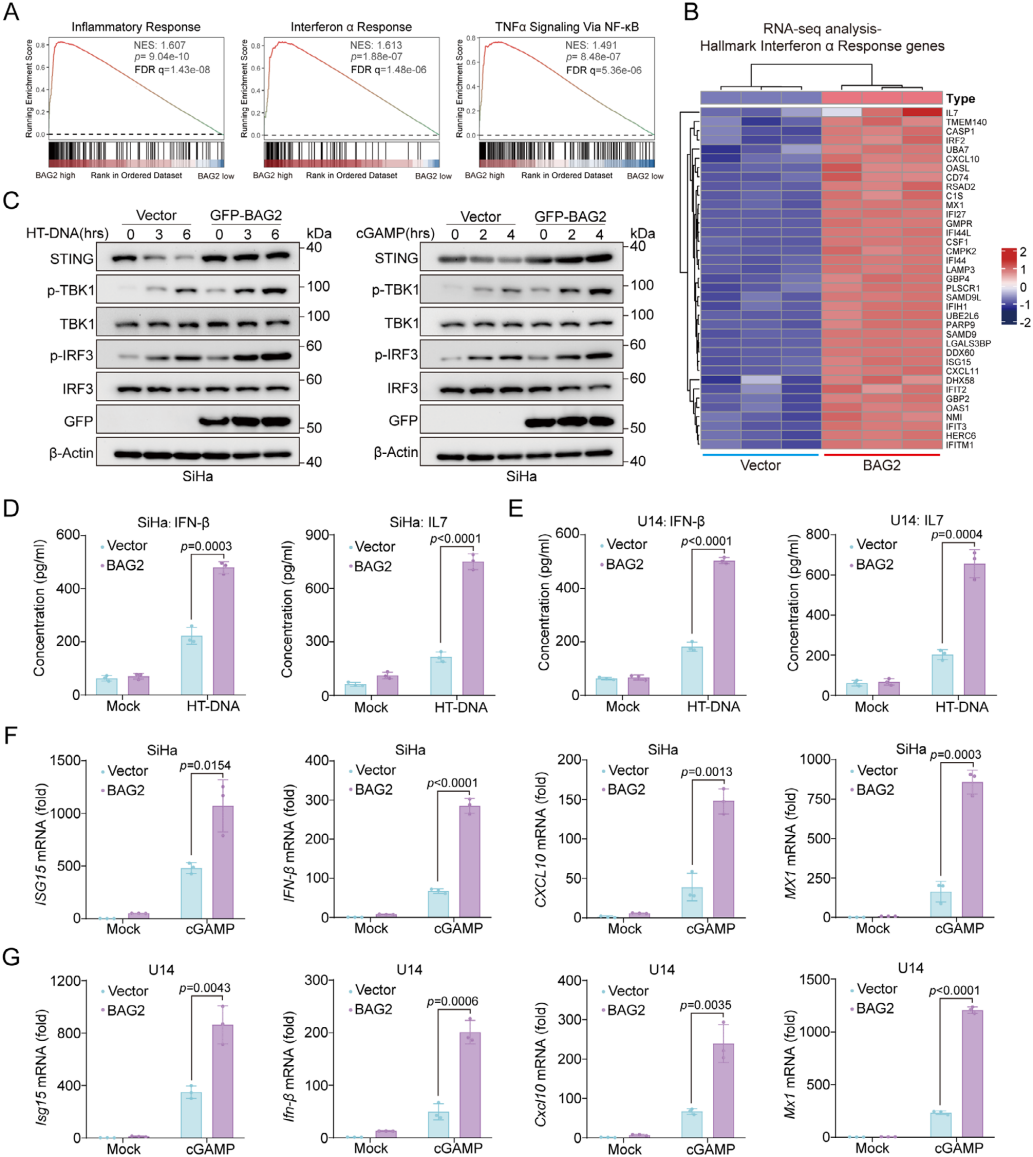
BAG2 positively regulates STING‐mediated type I interferon responses. A) RNA seq data of SiHa cells after BAG2 overexpression were analyzed by gene set enrichment analysis (GSEA), and Hallmark gene sets were used as annotated gene sets. B) Gene heat map of Hallmark Interferon α Response gene set after BAG2 overexpression in SiHa cells. C) SiHa cells were transfected with vector or GFP‐BAG2 for 48 h, and then they were treated with HT‐DNA for 0, 3, 6 h or cGAMP for 0, 2, 4 h. Cells were collected for detection of STING, p‐TBK1, TBK1, p‐IRF3, and IRF3 protein levels by Western blot. D) Vector or BAG2 plasmid was transfected in SiHa cells for 48 h, cell supernatants were collected by HT‐DNA treatment for 6 hrs, and IFN‐β and IL7 production was measured by ELISA. E) Vector or BAG2 plasmid was transfected in U14 cells for 48 h, cell supernatants were collected by HT‐DNA treatment for 6 h, and IFN‐β and IL7 production was measured by ELISA. F) SiHa cells and G) U14 cells were transfected with Vector or BAG2 for 48 h, then cGAMP was added for 4 h. qRT‐PCR was used to measure the mRNA expression of *ISG15*, *IFN‐β*, *MX1*, and *CXCL10*. Data are presented as mean values ± SD, with *n* = 3 (B, D‐G) biological independent experiments. Statistical significance was determined by two‐tailed unpaired Student's t‐test (D‐G).

To explore the potential mechanism of BAG2's downstream effects in cervical cancer, GSEA was performed using the TCGA cervical cancer dataset. The analysis revealed significant enrichment of the HALLMARK_INTERFERON_ALPHA_RESPONSE pathway (NES = 1.81, *p* = 0.009) in the BAG2 high‐expression group (Figure S5A, Supporting Information). Given that STING serves as the critical adaptor in type I interferon signaling, we hypothesized that BAG2 may be involved in regulating the type I interferon response. To test this hypothesis, BAG2 was overexpressed in SiHa cells, followed by RNA‐seq analysis. Interestingly, our data revealed that the BAG2 high‐expression group exhibited significantly elevated levels of several biological processes compared to the control, including Inflammatory Response (NES: 1.607, *p* = 9.04e‐10), Interferon α Response (NES: 1.613, *p* = 1.88e‐07) and TNFα Signaling Via NF‐κB (NES: 1.491, *p* = 8.48e‐07) (**Figure** **6**A). Notably, the BAG2 overexpression group demonstrated a marked increase in interferon‐stimulated genes (ISGs), such as ISG15, MX1, IFITM1, OASL, and OAS1 (Figure 6B), which are known to facilitate CD8^+^ T cell recruitment to tumor sites.^[^ ^31^ ^]^ This finding aligns with the role of interferon signaling in immune cell recruitment and the enhancement of anti‐tumor responses.^[^ ^32^ ^]^ These results suggest that BAG2 may modulate STING‐mediated type I interferon responses.

Next, we experimentally explored whether BAG2 activates the STING‐mediated type I interferon signaling pathway. It is commonly known that activation of the STING signaling pathway induces recruitment and phosphorylation of TBK1 which in turn triggers the IRF3 phosphorylation signaling cascade. As expected, BAG2 overexpression in SiHa cells resulted in enhanced phosphorylation of TBK1 and IRF3 in response to HT‐DNA and cGAMP (Figure 6C). To further substantiate this, ELISA analysis of cell supernatants revealed that BAG2 overexpression significantly increased the production of IFN‐β and IL7 (Figure 6D; Figure S5C, Supporting Information). Additionally, BAG2 overexpression induced the expression of *IFN‐β*, *MX1*, *CXCL10*, and *ISG15* in SiHa cells (Figure 6F; Figure S5E, Supporting Information). Overexpression of BAG2 in murine‐derived cervical cancer U14 cell line and obtaining the same results when stimulated with HT‐DNA and cGAMP (Figure 6E,G; Figure S5B,D,F, Supporting Information), suggesting that the function of BAG2 in regulating type I interferon responses is not species‐specific. To improve the generalizability and reliability of the conclusion, we also overexpressed BAG2 in HeLa cells and detected the phosphorylation levels of TBK1 and IRF3. Consistently, BAG2 overexpression enhanced HT‐DNA and cGAMP‐induced TBK1 and IRF3 phosphorylation (Figure S6A, Supporting Information). In addition, ELISA and qRT‐PCR analyses further validated that BAG2 overexpression promoted the production of ISGs in HeLa cells (Figure S6B–D, Supporting Information). Collectively, these results demonstrate that BAG2 positively regulates the STING‐mediated type I interferon signaling pathway.

### 2.7 BAG2 Inhibits Cervical Cancer Proliferation and Migration in a STING‐Dependent Manner

**Figure 7:**
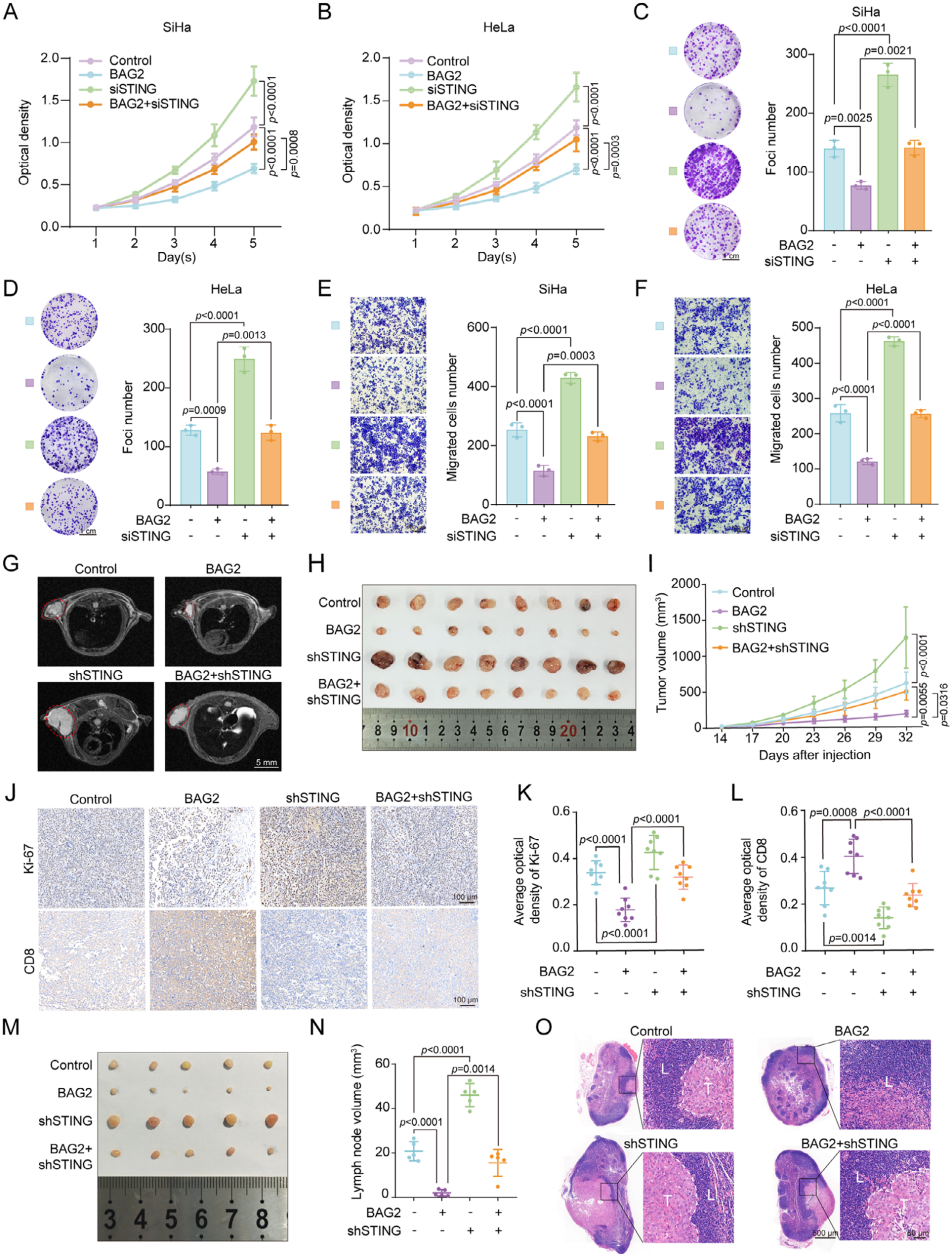
BAG2 inhibits cervical cancer proliferation and migration in a STING‐dependent manner. A,B) The proliferation capacity of SiHa cells and HeLa cells after BAG2 overexpression and/or *STING* knockdown was detected by CCK8 assay. C,D) Foci formation assay of SiHa cells and HeLa cells proliferation capacity after BAG2 overexpression and/or *STING* knockdown (left panel), followed by statistical analysis (right panel). E,F) Transwell assay showing migratory capacity of SiHa cells and HeLa cells after BAG2 overexpression and/or *STING* knockdown (left panel), followed by statistical analysis (right panel). G) MRI scans of various groups of subcutaneous tumors. H) Anatomical diagram of subcutaneous tumor tissue in different groups. I) Statistical plot of subcutaneous tumor growth volume between groups. J) IHC staining of subcutaneous tumor tissue for the tumor proliferation marker Ki67 and the T‐cell infiltration degree response marker CD8. K,L) IHC staining statistics of Ki‐67 and CD8 in subcutaneous tumor tissues. M) Anatomical images of popliteal lymph node metastasis model in control, BAG2, *shSTING* and BAG2+*shSTING* groups. N) Statistical diagram of popliteal lymph node volume between groups. O) H&E staining was used to analyze the tumor infiltrating foci of popliteal lymph nodes in mice. T = metastasized tumor tissue, L = normal lymph node tissue. Data are presented as mean values ± SD, with *n* = 3 (C‐F), 5 (N), 6 (A‐B), or 8 (I, K‐L) biological independent experiments. Statistical significance was determined by one‐way ANOVA with Tukey's multiple comparisons test (A‐F, I, K‐L, N).

Given that BAG2 is involved in STING‐mediated regulation of type I interferon signaling in cervical cancer cells, we continued to explore the role of the BAG2‐STING axis in anti‐tumor activity. We knocked down *STING* in BAG2 overexpressing SiHa and HeLa cells, and verified the protein levels of BAG2 and STING by Western blot (Figure S7A, Supporting Information). CCK8 assays showed that BAG2 overexpression significantly inhibited the proliferation of SiHa and HeLa cells, but this inhibition was counteracted in *STING* knockdown cells (**Figure** **7**A,B). Consistent with these results, foci formation assays showed that *STING* knockdown counteracted the inhibitory effect of BAG2 overexpression on the proliferative activity of SiHa and HeLa cells (Figure 7C,D). Moreover, transwell assays demonstrated that BAG2‐mediated inhibition of cell migration was dependent on STING (Figure 7E,F). Conversely, STING overexpression counteracted the proliferative and migratory effects induced by *BAG2* knockdown in SiHa and HeLa cells (Figure S7A–G, Supporting Information). To investigate whether the tumor suppressor effect of the BAG2‐STING axis is confined to HPV‐positive cells (HeLa: HPV18‐positive and SiHa: HPV16‐positive), additional functional assays were performed in HPV‐negative cells (C33A cells). We first verified the protein levels of STING and BAG2 by Western blot in C33A cells (Figure S8A, Supporting Information). CCK8 assays and foci formation assays showed that *STING* knockdown also reversed the inhibitory effect of BAG2 overexpression on the proliferative activity of C33A cells, whereas STING overexpression reverted the promotional effect of *BAG2* knockdown on the proliferative activity of C33A cells (Figure S8B–E, Supporting Information). Furthermore, transwell assays also demonstrated the inhibitory effect of the BAG2‐STING axis on the migration function of C33A cells (Figure S8F,G, Supporting Information). In summary, the BAG2‐STING axis exerted significant tumor suppressor effects in both HPV‐positive and HPV‐negative cervical cancer cells.

To determine the combined effects of STING and BAG2 on cervical cancer cell growth and metastasis in vivo, we constructed control, BAG2, *shSTING*, and BAG2+*shSTING* stably transfected U14 cell line, and then verified the protein levels of BAG2 and STING by Western blot (Figure S9A, Supporting Information). Subcutaneous tumor‐bearing experiments showed that the tumors in the BAG2 group grew slowly, whereas the tumor growth rate was significantly enhanced in the *shSTING* group. Notably, *shSTING* successfully counteracted the inhibitory effect caused by BAG2 overexpression (Figure 7G–I; Figure S9B, Supporting Information). IHC staining showed that *STING* knockdown significantly reverted the inhibitory effect of BAG2 overexpression group on Ki67 level (Figure 7J,K; Figure S9C, Supporting Information). In addition, we observed that BAG2 overexpression increased CD8^+^ T cell infiltration and that knockdown of *STING* counteracted the infiltrative effect of CD8^+^ T cells (Figure 7L). Since U14 cells are HPV‐negative, we constructed stable transfected cells in HPV‐positive TC‐1 cell line and performed subcutaneous tumor bearing experiments (Figure S9D, Supporting Information). The experimental results showed that *shSTING* effectively reversed the subcutaneous tumor suppression induced by BAG2 overexpression (Figure S9E–G, Supporting Information). These results further demonstrated the important role of the BAG2‐STING axis in tumor suppression, both for HPV‐positive and HPV‐negative tumor cells. Furthermore, we constructed a popliteal lymph node metastasis model using U14 cells and showed that BAG2 overexpression greatly inhibited tumor migration in vivo, and that this effect could be reversed by depletion of *STING* (Figure 7M,N). H&E staining confirmed the presence of tumor lymphatic metastases (Figure 7O). Thus, we demonstrated that BAG2 inhibits the proliferation and migration of cervical cancer cells by regulating STING, and this effect is present in both HPV‐positive and HPV‐negative tumor cells.

### 2.8 The Tumor Suppressor Function of the BAG2‐STING Axis is Only Partially Dependent on the STING‐HPV E7 Pathway

Recent studies have shown that activation of STING/TBK1 inhibits tumor growth by degrading the HPV16/18 E7 oncoprotein in cervical cancer.^[^ ^14^ ^]^ Here, we wished to explore whether the BAG2‐STING axis exerts tumor suppressive effects by affecting the degradation of HPV E7 oncoproteins. By Western blot analysis we found that *BAG2* knockdown up‐regulated the protein level of HPV E7 and down‐regulated the protein level of pRb, while STING overexpression significantly inhibited this effect, confirming the effectiveness of the BAG2‐STING axis on HPV E7 degradation (Figure S10A,B, Supporting Information). In addition to inactivating the oncogene pRb in virus‐infected cells, alterations in other signaling pathways are equally important for the transformation of HPV E7 oncogene‐transduced cells.^[^ ^33^ ^]^ PI3K/AKT/mTOR signaling pathway is frequently amplified in HPV‐induced cancers and plays a very important role in the oncogenic process.^[^ ^34^ ^]^ We found that *BAG2* knockdown activated the AKT/mTOR signaling pathway, which was inhibited by STING overexpression or *HPV E7* silencing (Figure S10A,B, Supporting Information). Thus, our findings suggest that the BAG2‐STING axis influences AKT/mTOR pathway activation through regulation of HPV E7 degradation.

Interestingly, cell proliferation and migration function assays demonstrated that although *HPV E7* knockdown could partially recover the increased proliferation and migration ability of cervical cancer cells caused by *BAG2* knockdown, its effect was significantly lower than the recovery ability of STING overexpression (Figure S10C–H, Supporting Information). In view of these results and the important role that STING plays in innate immunity, it is reasonable to assume that STING‐mediated degradation of HPV E7 is only a part of the effect of the BAG2‐STING axis on the development of cervical cancer, and that BAG2‐STING inhibits the progression of cervical cancer mainly through type I interferon signaling.

**Figure 8:**
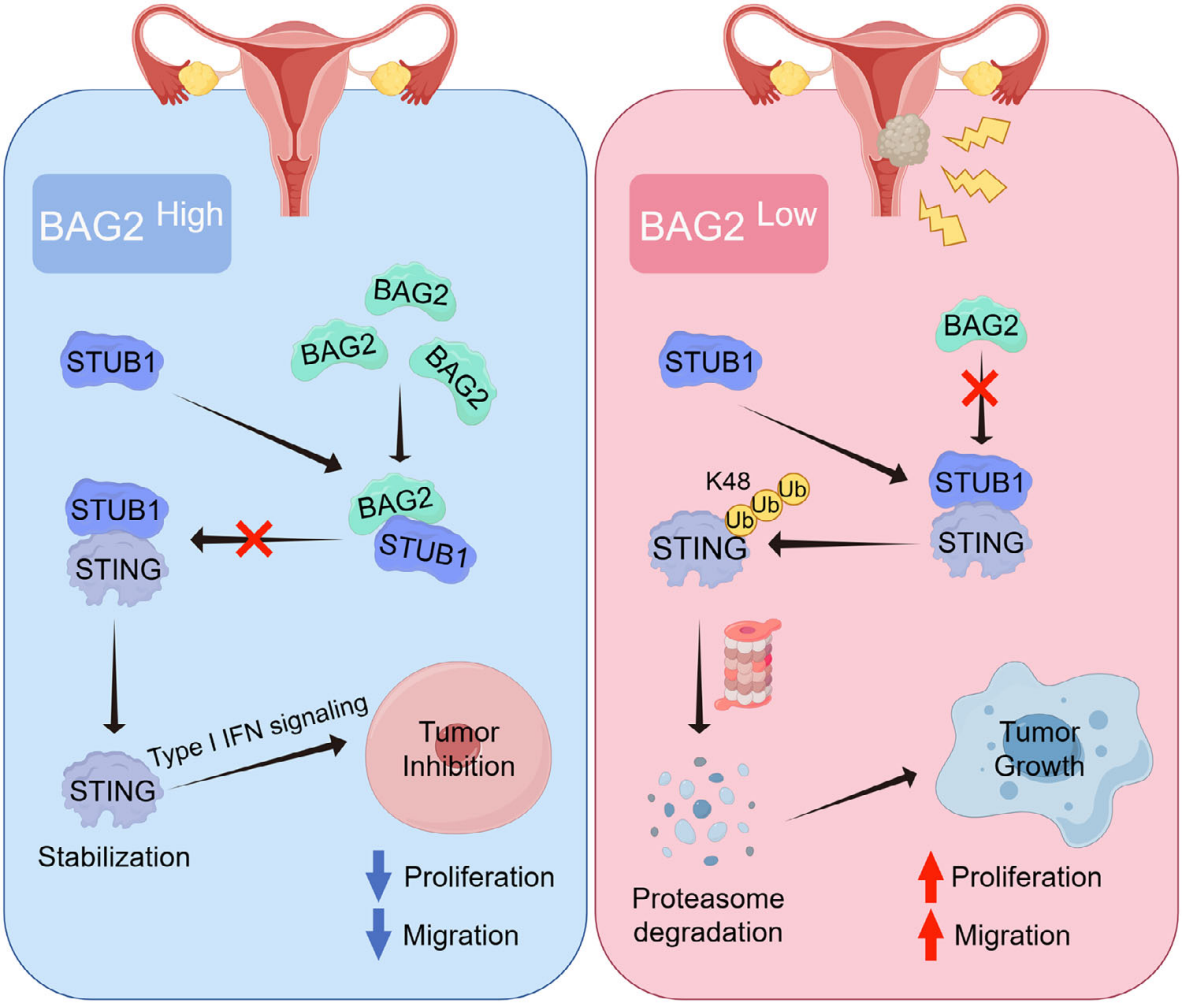
Mechanism diagram of the study. The mechanism diagram illustrates the BAG2‐STING axis that inhibits the proliferation and metastasis of cervical cancer. BAG2 inhibits the degradation of STING through the E3 ubiquitin ligase STUB1, thereby inhibiting the progression of cervical cancer. Specifically, BAG2 inhibits STUB1 from attaching the K48‐linked ubiquitin chains at K338 and K370 of STING by forming a complex with STUB1, thereby enhancing its protein stability and activating the STING‐mediated type I interferon signaling pathway. This Figure was drawn by an online tool Figdraw (https://www.figdraw.com/static/index.html#/).

In summary, BAG2 was identified as a key regulator of STING protein stability, where it forms a complex with STUB1 to inhibit K48‐linked ubiquitination at the K338 and K370 sites of STING, thus enhancing its stability. Moreover, BAG2 controls cervical cancer cell proliferation and migration and inhibits cancer progression by modulating STING‐mediated type I interferon signaling (**Figure** **8**).

## 3 Discussion

The advent of immunotherapy has ushered in new possibilities for cancer treatment, with immunotherapeutic agents now included in various treatment guidelines for both adjuvant and neoadjuvant therapies. Despite these advances, a significant proportion of patients still do not respond adequately to immunotherapy.^[^ ^35^ ^]^ Current research is focused on identifying novel targets that could trigger or enhance anti‐tumor immune responses. Among these, STING has garnered substantial attention as a key sensor in the innate immune response. Studies have shown the critical involvement of the STING in endogenous tumor suppression and cancer immunity.^[^ ^36^ ^]^ Recent advancements in STING agonist research have demonstrated their potential to enhance anti‐tumor responses in different cancer models, such as hepatocellular carcinoma,^[^ ^37^ ^]^ ovarian cancer,^[^ ^38^ ^]^ and colorectal cancer.^[^ ^39^ ^]^ However, to evade immune detection, cancer cells often impair or lose the function of the STING signaling pathway, thereby conferring resistance to STING agonists.^[^ ^40^ ^]^ Consequently, identifying strategies to stabilize STING proteins and finding potential targets that can augment the anti‐tumor activity of STING remain pivotal areas of cancer immunotherapy research. In this study, we reveal the critical role of the chaperone protein BAG2 in the regulation of STING protein stability. Specifically, BAG2 activates the STING‐mediated type I interferon signaling pathway by inhibiting STING ubiquitination and degradation, thereby suppressing cervical carcinogenesis and progression.

Accumulating evidence indicates that the therapeutic efficacy of many anti‐tumor approaches, including immunotherapy, is largely dependent on type I interferon signaling.^[^ ^41^ ^]^ As a key transducer in innate immunity, STING detects cytoplasmic DNA abnormalities, which phosphorylates TBK1 and IRF3, which subsequently triggers the production of type I interferon and pro‐inflammatory cytokines. In the present study, we identified BAG2 as a positive regulator that induces STING‐associated type I interferon signaling activation. Specifically, RNA‐seq results revealed that BAG2 is associated with the expression of multiple ISGs in cervical cancer cells, which are vital for anti‐tumor immunity as an IFN‐induced gene. Further, GSEA revealed that cervical cancer cells with BAG2 overexpression was significantly enriched in the type I interferon signaling pathway. Stimulation of the STING‐related type I interferon pathway is closely linked to enhanced anti‐tumor T cell infiltration.^[^ ^42^ ^]^ Notably, the activation of STING signaling pathway is known to sustain CD8^+^ T cell infiltration and promote anti‐tumor immunity, and STING pathway activation is implicated in mediating the clearance of drug‐resistant tumor cells by CD8^+^ T cells.^[^ ^13^, ^43^ ^]^ The present study demonstrates that BAG2 inhibits the ubiquitination of STING, thereby stabilizing the protein and activating the STING‐TBK1‐IRF3 signaling pathway, which promotes the production of type I interferon genes and downstream chemokines. This finding prompted us to further explore whether BAG2 is associated with tumor infiltration of CD8^+^ T cells. Indeed, in our subcutaneous tumor model, a significant increase in CD8^+^ T cell infiltration was observed in the BAG2 overexpression group. It is suggested that BAG2 may have a broader immunomodulatory function in cervical cancer.

Ubiquitination and deubiquitination are key regulatory mechanisms for STING activity.^[^ ^44^ ^]^ For instance, RNF144A interacts with STING and promotes its K6‐ linked ubiquitination at the K236 site.^[^ ^45^ ^]^ TRIM10 is an E3 ubiquitin ligase that binds to STING and catalyzes the multiubiquitination of K27‐ and K29‐ linked STING at K289 and K370 sites.^[^ ^46^ ^]^ In the present study, the BAG structural domain of BAG2 interacts with the TM1 transmembrane domain of STING. BAG2 inhibits the formation of K48‐linked ubiquitin chain on STING at K338 and K370 sites, a process facilitated by BAG2's suppression of the E3 ligase STUB1. Moreover, we demonstrated a significant positive correlation between BAG2 and STING protein levels by analyzing cervical cancer clinical samples. In addition, we found that mRNA and protein levels of BAG2 were significantly reduced in cervical cancer tissues compared with paracancerous tissues. Importantly, patients with high BAG2 protein level had better overall survival outcomes and significantly lower tumor recurrence rates. Lymph node metastasis is the main mode of metastasis for cervical cancer, and its presence often correlates with poorer patient prognosis.^[^ ^47^ ^]^ Using a mouse popliteal lymph node metastasis model, this study demonstrated that the BAG2‐STING axis significantly inhibits cervical cancer lymph node metastasis in vivo. The relevance of this regulatory axis to cervical cancer development was further confirmed through advanced clinical imaging techniques, including MRI and PET/CT scans. These results suggest that BAG2 may be a promising therapeutic target for cervical cancer.

STING was shown to inhibit cervical cancer progression by degrading HPV16/18 E7.^[^ ^14^ ^]^ We found that the BAG2‐STING axis altered the levels of HPV E7 and its downstream pRb and AKT signaling pathway‐related protein. However, *HPV E7* knockdown did not completely reverse the *BAG2* knockdown‐induced enhancement of Hela and Siha cell proliferation and migration compared with STING overexpression. This suggests that STING‐mediated degradation of HPV E7 is only a part of the BAG2‐STING axis effect on cervical cancer development, and that BAG2 regulation of HPV E7 proteins may be through a cumulative effect of stabilizing STING protein level. Consistent with this, cellular experiments showed that STING effectively reversed the changes in tumor activity induced by *BAG2* knockdown or overexpression and was independent of HPV infection status. This conclusion was further supported by subcutaneous tumor‐bearing experiments. That is, the tumor suppressor effect of the BAG2‐STING axis has broad applicability in cervical cancer cells under different HPV infection status.

To sum up, our findings show that BAG2 is a positive regulator of STING‐associated type I interferon signaling in cervical cancer. We also clarify that BAG2 stabilizes STING by preventing STING from being ubiquitinated by STUB1, thereby preventing cervical cancer proliferation and metastasis. The BAG2‐STING axis plays a pivotal role in the development of cervical cancer, and targeting BAG2 may be a potential therapeutic strategy for cervical cancer.

## 4 Experimental Section

### Human Tissues

Human cervical cancer and paraneoplastic tissue samples were collected from the Department of Gynecologic Oncology, Zhongnan Hospital, Wuhan University, Wuhan, China (approval number: 2020029), and informed consent was obtained from all individuals. Postoperative pathological examination confirmed their pathological type. Patients undergoing immunotherapy for cervical cancer had cervical tissue removed by biopsy prior to immunotherapy, and patient responsiveness to immunotherapy was assessed by the immune response evaluation criteria in solid tumors (iRECIST). The cervical cancer tissue microarray and corresponding clinical data (HUteS168Su01, containing 119 tumor tissues and 39 paraneoplastic tissues) were provided by Shanghai Outdo Biotech Co., Ltd. (http://www.superchip.com.cn/index.html).

### Cell Culture and Transfection

American Type Culture Collection (ATCC, USA) provided and identified the HeLa, SiHa, C33A, CaSki, HaCaT, U14, TC‐1, and HEK293T cells utilized in this study. HeLa, SiHa, C33A, U14, HaCaT, and HEK293T cells were cultured in DMEM media supplemented with 10% fetal bovine serum. CaSki and TC‐1 cells were cultured in RPMI 1640 media supplemented with 10% fetal bovine serum. All cell lines were negative for mycoplasma infection. For transfection, Lipofectamine 3000 (L3000015, Invitrogen) was utilized.

### Antibodies and Compounds

The lists of antibodies and compounds are shown in Tables S6 and S7 (Supporting Information), respectively.

### siRNA and Plasmids

*siBAG2* (#1: 5’‐GAAAUCCUUCUGGAAAUGA‐3’, #2: 5’‐CCGUUUGAUGGGAAGAACU‐3’; #3: 5’‐CAGUUGAUCAGAAGUUUCA‐3’), *siSTUB1* (#1: 5’‐ ACCACGAGGGUGAUGAGGA‐3’, #2: 5’‐ GCAGUCUGUGAAGGCGCACU‐3’;), *siSTING* (5’‐ GCACCUGUGUCCUGGAGUA‐3’), *siHPV16 E7* (5’‐CACCTACATTGCATGAATA‐3’) and *siHPV18 E7* (5’‐CCTTCTATGTCACGAGCAA‐3’) were purchased from Tsingke Biotech Co., Ltd. (Beijing, China). GFP‐BAG2, Flag‐BAG2, HA‐STUB1, Flag‐STING, GFP‐BAG2 (amino acids 1–100), GFP‐BAG2 (amino acids 101–211), Flag‐STING (amino acids 1–139), Flag‐STING (amino acids 140–379) and Myc‐Ub were purchased from Tsingke Biotech Co., Ltd. (Beijing, China). Flag‐STING‐ΔTM1 (G17_E41del), Flag‐STING‐ΔTM2 (R45_E69del), Flag‐STING‐ΔTM3 (G90_G114del), Flag‐STING‐ΔTM4 (P115_L139del), Flag‐STING (K20R, K137R, K150R, K224R, K236R, K289R, K338R, K370R, L30A, W34A, L51A, Q55A, L58A, L139A, and L30A/W34A) and GFP‐BAG2 (I188A, E192A, S194A, G198A, N205A, F210A, and I188A/E192A) plasmids were constructed in this laboratory by standard subcloning and their integrity was determined by DNA sequencing.

### Quantitative Reverse Transcription PCR (qRT‐PCR)

Following the manufacturer's instructions, total RNA was extracted from the cells using TRIzol reagent (15596018CN, Invitrogen). HiScript Q Select RT SuperMix (R233‐01, Vazyme Biotech) was then used to reverse transcribe the extracted RNA in order to create cDNA samples. SYBR qPCR premix (Q711‐03, Vazyme Biotech) was used for the quantitative real‐time PCR, and the results were quantified using CFX real‐time PCR detection equipment (BIO‐RAD, USA). The sequences of primers used are listed in Table S8 (Supporting Information).

### Immunofluorescence

Cells were placed in 6‐well plates fitted with sterilized 22 × 22 mm coverslips. After fixation with paraformaldehyde and washing, cells were incubated in 2% BSA and 0.3% Triton X‐100 solution for 40 min at room temperature. Cells were incubated with specific primary antibody for one night at 4 °C, followed by incubation with DAPI and fluorescent secondary antibody for 2 h at room temperature. Finally, photographs were taken using a laser confocal microscope (Nikon, Japan).

### Immunohistochemistry (IHC)

Samples underwent a series of procedures including formalin fixation, paraffin embedding, sectioning, deparaffinization, antigenically corrected, and blocking endogenous peroxidase. After the specified primary and secondary antibodies were added to the slides, a fresh DAB color development solution was added. Finally, a multifunction scanning microscope was used to take pictures of the slides.

### Flow Cytometry

SiHa cells were transfected, then centrifuged and given two rounds of cold PBS washing. Following that, cells were suspended in permeabilization solution and propidium iodide (PI, 100 µg mL^−1^) from the Cell Cycle Staining Kit (CCS012, Multi sciences) and incubated for 30 min at room temperature in the dark. FlowJo software (version 7.6) was used to process the data after samples were analyzed using a Beckman Cytoflex flow cytometer.

### Western Blot Analysis

Proteins were denatured at 100 °C after lysing cells on ice for 30 min using a mixture of RIPA buffer, PMSF, and phosphatase inhibitor. After being separated on an SDS‐PAGE gel, denatured proteins were transferred to a PVDF membrane. After blocking the PVDF membrane with 5% skim milk, the membrane was incubated with specific primary antibodies and matched secondary antibodies. Ultimately, chemiluminescence was used to detect the level of the protein.

### Co‐Immunoprecipitation (Co‐IP) Assay

Co‐IP were performed using the BeaverBeads Protein A/G Immunoprecipitation Kit (22202‐100, Beaver). Briefly, cells were incubated with MG132 (10 µm, S2619, Selleck) for 6 h prior to collection, and then samples were prepared in cell lysate buffer containing PMSF and phosphatase inhibitors, and then incubated with the target antibody overnight at 4 °C. After the sample mixture was incubated with protein A/G beads for 2 h at 4 °C, the immunoprecipitates were washed three times and 1x SDS buffer was added. The proteins were denatured by applying heat and separated from the magnetic beads. Ultimately, Western blot was used to determine the protein concentration.

### GST Pull‐Down Assay

Recombinant GST‐tagged STING and His tagged BAG2 and STUB1 were purified from *Escherichia coli* BL21. GST‐STING fusion protein was incubated with His‐BAG2 and His‐STUB1 fusion proteins for 4 h at 4 °C, respectively. The mixtures were incubated with glutathione‐Sepharose beads for 3 h at 4 °C for GST pull‐down assay. GST pull‐down products were eluted with 1× SDS buffer and denatured at 100 °C for 6 min, then used for Western blot.

### Proximity Ligation Assay (PLA)

The cell processing procedure was consistent with immunofluorescence, and after overnight incubation with the corresponding primary antibodies, the samples were washed with wash buffer (DUO82049, Sigma). Secondary antibodies with PLA probes (anti‐mouse PLUS probe: DUO92001‐30RXN; anti‐rabbit MINUS probe: DUO92005‐30RXN, Sigma) were incubated with the samples for 2 h at room temperature. After washing the samples with washing buffer, the samples were incubated with Duolink In Situ Red Detection Reagent (DUO92008, Sigma) for 2 h at room temperature. Finally, the nuclei were stained with DAPI, and images were captured by laser confocal microscopy.

### Cycloheximide (CHX)‐Chase Assay

The cell culture media were supplemented with 50 µg mL^−1^ CHX (HY‐12320, MCE), and the cells were taken for Western blot analysis at the designated time intervals. ImageJ software was used to quantify the target protein levels.

### In Vivo Ubiquitination Assay

Before collecting the cells, 10 µm MG132 was added to the cell culture medium. The cells were then lysed using a solution of RIPA lysis buffer, PMSF, and phosphatase inhibitor, and the level of protein ubiquitylation was measured using the Co‐IP technique.

### In Vitro Ubiquitination Assay

STING, BAG2, and STUB1 proteins were expressed with the TnT Quick Coupled Transcription/Translation System kit (L1170, Promega) according to the manufacturer's instructions. In vitro ubiquitination assay was performed with the ubiquitination kit (BML‐UW9920‐0001, Enzo Life Science) following the manufacturer's protocols.

### Immunoprecipitation‐Mass Spectrometry (IP‐MS) Analysis

The Flag‐STING plasmid was transfected into SiHa cells for 48 h before the proteins were analyzed utilizing Co‐IP with anti‐IgG antibody as a negative control. The sample was separated using SDS‐PAGE and stained using Fast Silver Stain Kit (P0017S, Beyotime). Mass spectrometry was conducted by SpecAlly Life Technology Co., Ltd (Wuhan, China). All samples were evaluated using an UltiMate 3000 RSLCnano system coupled on‐line with Q Exactive HF mass spectrometer via a Nanospray Flex ion source (Thermo). MaxQuant was used to analyze raw MS data using the Andromeda database search algorithm.

### RNA‐seq and Bioinformatics Analysis

SiHa cells were transfected with Vector and BAG2 overexpression plasmids for 48 h. Total RNA was extracted using TRIzol reagent, concentration was checked for extracted nucleic acids using a Nanodrop (Thermo Fisher Scientific, USA), and integrity was checked using the Agilent 2100 Bioanalyzer (Agilent Technologies, USA). The libraries were then constructed using the VAHTS Universal V6 RNA‐seq Library Prep Kit according to the manufacturer's instructions. Transcriptome sequencing and analysis were performed by Tsingke Biotech Co (Beijing, China). Gene set enrichment analysis (GSEA) was performed on 50 cancer hallmark gene sets using the R‐package “clusterProfiler”. q‐value < 0.05 was considered statistically significant.

### Proliferation Experiment

To test cell viability in the CCK8 assay, 3000 transfected cells were inoculated into each well of 96‐well plates. Subsequently, 10 µL of CCK‐8 solution was injected to each well at different time intervals, and the absorbance at 450 nm was recorded.

Transfected cells were cultivated in 6‐well plates at a density of 1000 cells per well for foci formation assay, and they were incubated for around two weeks at 37 °C. After discarding the media, the cells were fixed for 30 min with 4% formaldehyde, stained for 30 min with 0.1% crystal violet, rinsed with water, and dried.

### Migration Experiment

For transwell assays, 200 µL of serum‐free media was combined with 40,000 cells, which were then injected into the top chamber (Corning, USA). Subsequently, the bottom chamber was filled with 600 µL of serum‐containing medium. The cells were incubated for 24 h, fixed for 30 min, and then stained for 1 h using crystal violet. Following washing and drying, the cells were counted using ImageJ software.

### ELISA Assay

After transfecting the cells, the supernatant was collected, centrifuged to remove the precipitate, and the samples were processed according to the instructions of IL7 kit (EK207, EK107, MultiSciences Biotech) and IFN‐β kit (EK2236, EK1236, MultiSciences Biotech), and then the absorbance value was detected by a microplate reader.

### Animal Studies

The animal study was approved by Zhongnan Hospital of Wuhan University's Experimental Animal Welfare Ethics Committee (approval number: ZN2024081). Female BALB/c mice were bought that were 4 weeks old from GemPharmatech Co., Ltd (Jiangsu, China). Lentivral transfection of U14 cells and TC‐1 cells were used in animal experiments. GenePharma (Shanghai, China) supplied the lentiviral, and puromycin (1 µg mL^−1^, Sigma,) was used to screen the transfection.

In the subcutaneous tumor‐bearing model, mice received a subcutaneous injection of 1 × 10^7^ U14 cells or TC‐1 cells. The tumor size was evaluated using the following formula, which was recorded every three days starting from the second week following the injection: tumor size (mm^3^) = (length × width^2^)/2. Subcutaneous tumor tissues were imaged using a 7.0T BioSpec 70/20 (Bruker, USA) small animal magnetic resonance imager (MRI). For positron emission tomography computed tomography (PET/CT) imaging, mice were injected with 18F‐FDG (150 µCi/per mouse) in the tail vein, anesthetized with 2% isoflurane 1 h later, and then subjected to a 10 min static PET scan using an Inliview‐3000B system (Novelmedical, China). Data were reconstructed using a 3D ordered subsets expectation maximization algorithm. The mice were then euthanized, the subcutaneous tumors were excised, and the tumor tissue was carefully dissected, weighed, and photographed. H&E and IHC were used to stain the tumor tissues.

For the popliteal lymph node metastasis model, 1 × 10^6^ U14 cells that had been resuspended in 50 µL of sterile PBS and injected into the right footpads of mice. Mice were observed twice a week, and then were euthanized after 4 weeks, and the popliteal lymph nodes were dissected, measured and fixed, and stained with H&E.

### Molecular Docking Analysis

The protein models used for docking were BAG2 (UniProt ID: O95816) and STING (UniProt ID: Q86WV6). Protein‐protein molecular docking was carried out using the HDOCK server (http://hdock.phys.hust.edu.cn), and the model with the highest score was determined to be the best docking model. Lastly, Pymol 2.4 software was used to visualize the docking results.

### Statistical Analysis

The statistical analyses were carried out with version 9.0 of GaphPad Prism. The two‐tailed Student's t‐test was employed to compare the two groups. One‐way ANOVA was used to evaluate multiple group differences, Tukey test was used to compare all column pairs, and Dunnett test was used to compare all columns with the control column. The Kaplan‐Meier technique was employed for survival analysis. *p* < 0.05 were judged as a statistical difference.

## Conflict of Interest

The authors declare no conflict of interest.

## Author Contributions

S.Y. and S.C. contributed equally to this work. S.Y. and H.C. designed and supervised the study. S.Y. and S.C. performed the most experiments. S.Y., S.C., Z.L., A.W., X.L., and Y.G. analyzed the results and collected the specimens. S.Y. and S.C. wrote the first draft. H.C. critically revised drafts of the manuscript. All authors reviewed the manuscript.

## Supporting information



Supporting Information

Supplemental Data 1

Supplemental Data 2

## Data Availability

The RNA‐seq data generated in this study have been deposited in the GEO database (https://www.ncbi.nlm.nih.gov/geo/) with the access code GSE279947. The mass spectrometry proteomics data generated in this study are provided in Supplementary Dataset 1. The remaining data are available within the article or Supporting Information. Original data are provided with this paper.
