# Supplementary material for: BAG2 Inhibits Cervical Cancer Progression by Modulating Type I Interferon Signaling through Stabilizing STING
Source: Adv Sci (Weinh). 2025 May 14;12(29):e70005. doi: 10.1002/advs.202414637 (PMC12362813; doi:10.1002/advs.202414637)
Supplement: Supplementary file 1 — Supporting Information [file ADVS-12-e70005-s003.pdf]

ADVANCED SCIENCE
Open Access

Supporting Information

for *Adv. Sci.*, DOI 10.1002/advs.202414637

BAG2 Inhibits Cervical Cancer Progression by Modulating Type I Interferon Signaling through Stabilizing STING

*Shijie Yao, Siming Chen, Anjin Wang, Ziyan Liang, Xuelian Liu, Yang Gao and Hongbing Cai**

Supplementary Information

BAG2 Inhibits Cervical Cancer Progression by Modulating Type I Interferon Signaling through Stabilizing STING

Figure S1-S11: Pages 2-15

Table S1-S8: Pages 16-25

Figure S1-S11

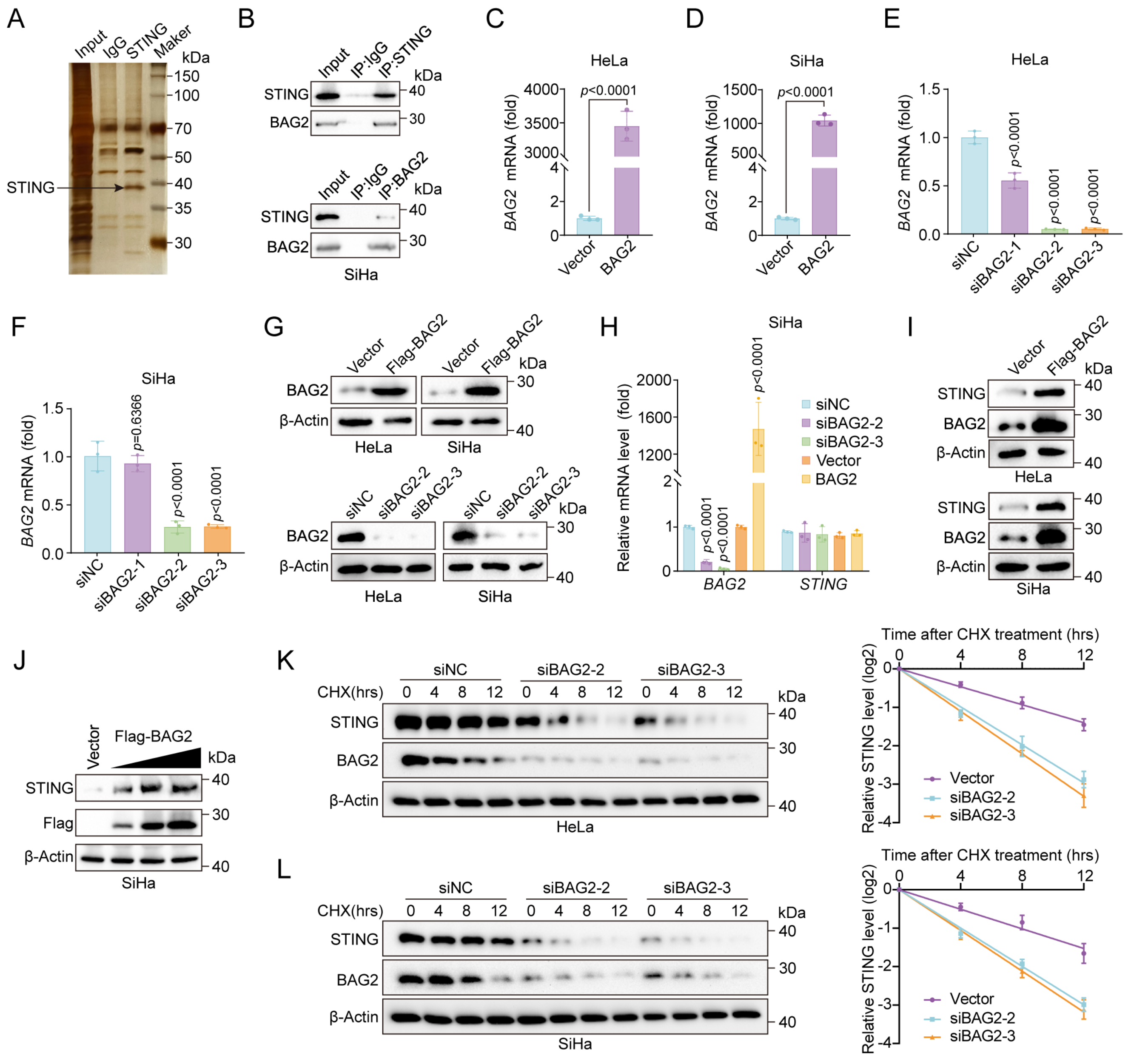

Figure S1. BAG2 binds and stabilizes STING.

(A) Silver staining of STING-interacting proteins in SiHa cells. **(B)** Western blot analysis of STING and BAG2 in SiHa cells after IP analysis with antibodies against STING (top panel) and BAG2 (bottom panel). **(C-D)** qRT-PCR to verify the overexpression level of BAG2 in HeLa and SiHa cells. **(E-F)** qRT-PCR validation of knockdown levels of *BAG2* in HeLa and SiHa cells. **(G)** The overexpression and knockdown levels of BAG2 in HeLa and SiHa cells were confirmed by Western blot analysis. **(H)** mRNA expression of *BAG2* and *STING* after BAG2 knockdown or overexpression were detected by qRT-PCR in SiHa cells. **(I)** After transfection of HeLa and SiHa cells with BAG2 overexpression plasmid, STING protein levels were detected by Western blot. **(J)** Western blot analysis of STING after the transfection of SiHa cells with Vector or Flag-BAG2 (0.2 µg, 1.0 µg or 4.0 µg). **(K)** 50 µg/mL cycloheximide (CHX) was used to treat HeLa cells transfected with *siBAG2*, and the cells were taken out at the designated intervals. Representative Western blot plots showing the impact of *BAG2* knockdown on STING degradation (left panel) and protein half-life statistics (right panel). **(L)** 50 µg/mL CHX was used to treat SiHa cells transfected with *siBAG2*, and the cells were taken out at the

designated intervals. Representative Western blot plots showing the impact of *BAG2* knockdown on STING degradation (left panel) and protein half-life statistics (right panel). Data are shown as mean ± SD, with n = 3 (C-F, H, K-L) biological independent experiments. Statistical significance was determined by two-tailed unpaired Student's t-test (C-D), or one-way ANOVA with Dunnett's multiple comparisons test (E-F). The one-way ANOVA with Dunnett's multiple comparisons test was utilized to ascertain statistical differences within the *BAG2* knockdown group, the two-tailed unpaired Student's t-test was used to assess the statistical difference for the BAG2 overexpression group (H).

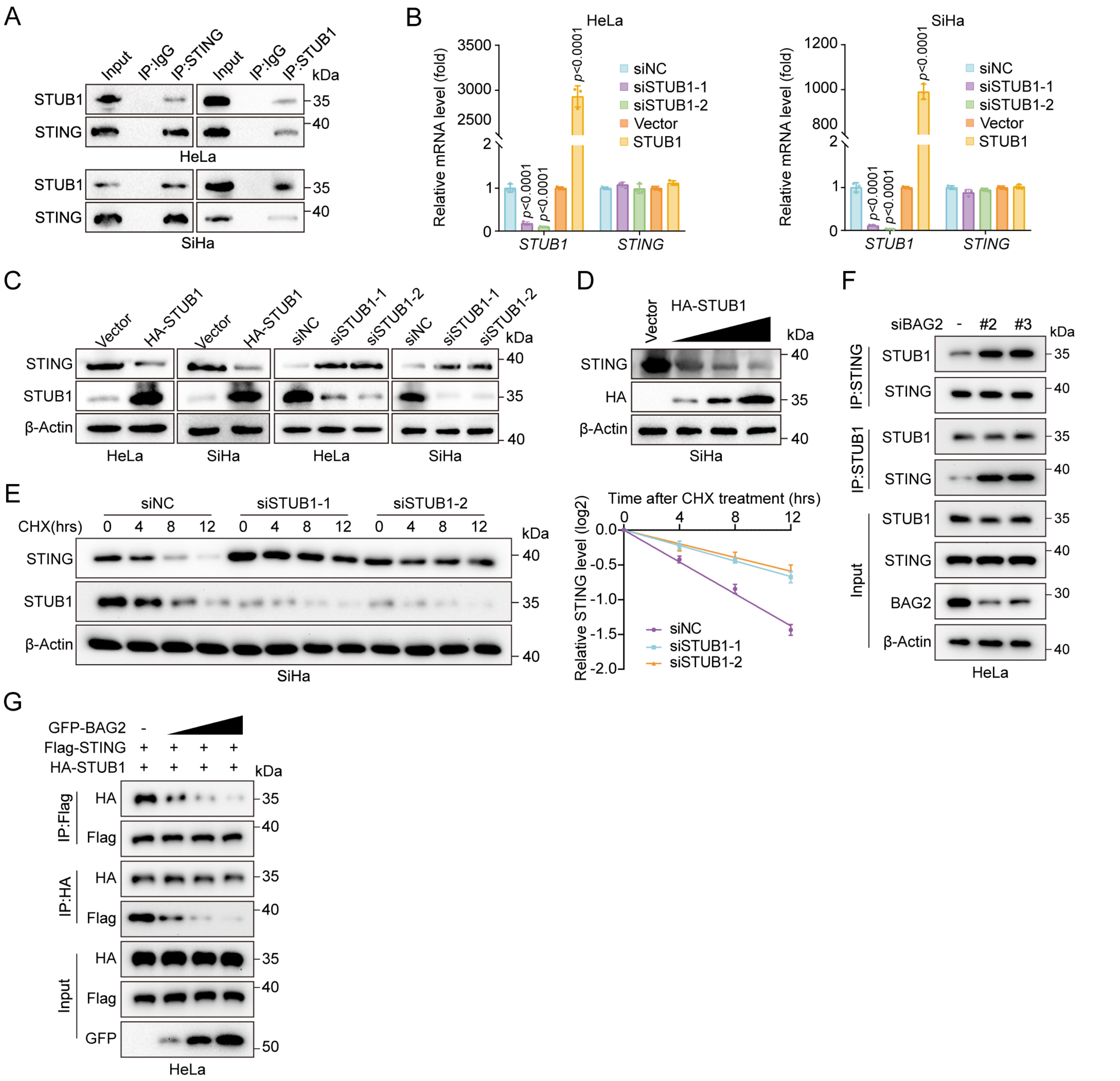

Figure S2. BAG2 regulates the stability of STING by STUB1.
(A) Western blot analysis of STING and BAG2 in HeLa cells (top panel) and SiHa cells (bottom panel) after IP analysis with antibodies against STING and BAG2. **(B)** mRNA expression of *BAG2* and *STING* after BAG2 knockdown or overexpression were detected by qRT-PCR in HeLa cells (left panel) and SiHa cells (right panel). **(C)** Western blot demonstrating STING protein levels following STUB1 overexpression or knockdown in HeLa and SiHa cells. **(D)** Western blot analysis of STING protein levels after the transfection of SiHa cells with Vector or HA-STUB1 (0.2 μg, 1.0 μg or 4.0 μg). **(E)** 50 μg/mL CHX was used to treat SiHa cells transfected with *siSTUB1*, and the cells were taken out at the designated intervals. Representative Western blot plots showing the impact of *STUB1* knockdown on STING degradation (left panel) and protein half-life statistics (right panel). **(F)** HeLa cells transfected with *siBAG2* were incubated with MG132 (10 μM) for 6 hrs, and Western blot analysis of STUB1 and STING was performed after IP analysis with STING and STUB1 antibodies. **(G)** HeLa cells transfected with Flag-STING, HA-STUB1 and GFP-BAG2 (0.2 μg, 1.0 μg, or 4.0 μg) were incubated with MG132 (10 μM) for 6 hrs, and Western blot analysis of HA-STUB1 and Flag-STING

was performed after IP analysis with Flag and HA antibodies. Data are shown as mean ± SD, with n = 3 (B, E) biological independent experiments. The one-way ANOVA with Dunnett's multiple comparisons test was utilized to ascertain statistical differences within the *STUB1* knockdown group, the two-tailed unpaired Student's t-test was used to assess the statistical difference for the STUB1 overexpression group (B).

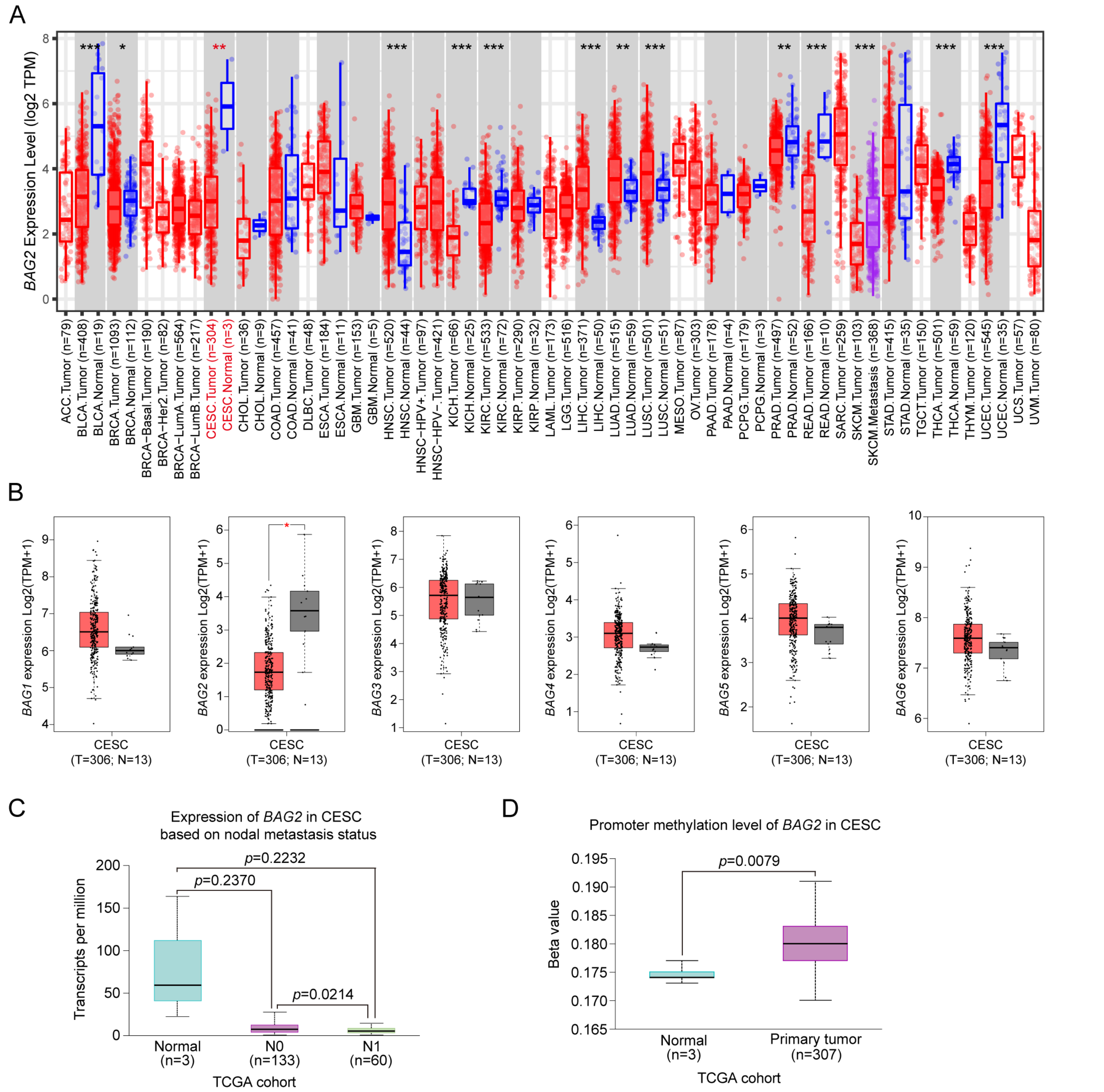

Figure S3. BAG2 is associated with prognosis in cervical cancer.

(A) Distribution of pan-cancer *BAG2* mRNA expression from the TIMER2 database (http://timer.comp-genomics.org/), x-axis indicates tumor type, y-axis indicates *BAG2* expression (log2 TPM). Red indicates the distribution of BAG2 in tumor tissues and blue indicates normal tissues. TPM: transcripts per million kilobases. **(B)** mRNA expression of BAG family members (*BAG1*, *BAG2, BAG3*, *BAG4*, *BAG5*, and *BAG6*) in TCGA-CESE, and the y-axis indicates *BAG* expression (log2(TPM+1)). **(C)** The UALCAN database showed *BAG2* expression levels in cervical cancer tissues of different stages (N0 and N1) as well as normal cervical tissues. **(D)** The UALCAN database showed *BAG2* promoter methylation levels in cervical cancer tissues and normal cervical tissues. Data are shown as mean ± SD. Statistical significance was determined by Wilcoxon test (A), two-tailed unpaired Student's t-test (B, D), or one-way ANOVA with Tukey's multiple comparisons test (C).

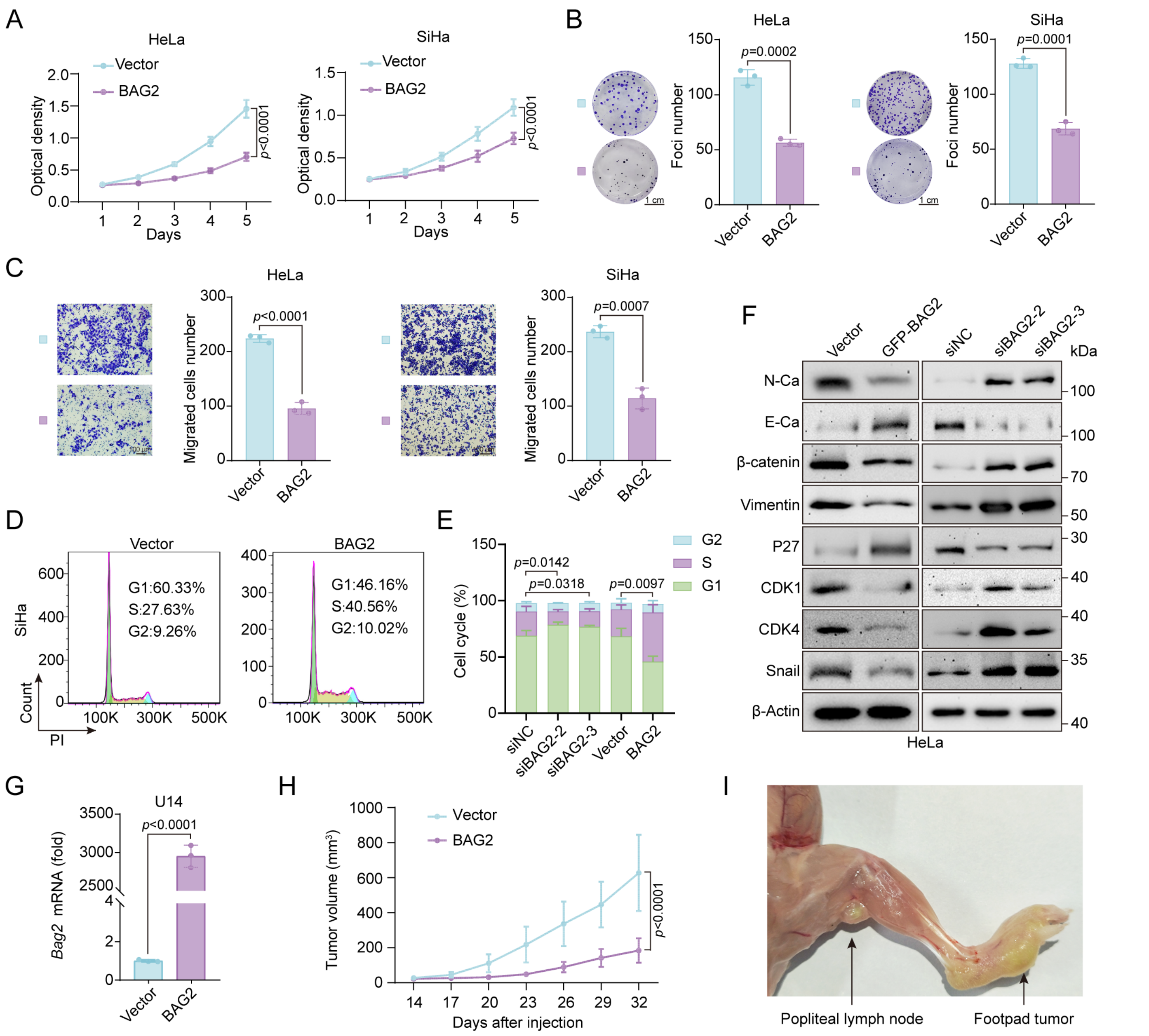

Figure S4. BAG2 inhibits cervical cancer progression both *in vivo* and *in vitro*.
(A) The proliferation capacity of SiHa and HeLa cells after BAG2 overexpression was determined by CCK8 assay. **(B)** Foci formation assay of proliferative capacity of SiHa and HeLa cells after BAG2 overexpression treatment (left panel), followed by statistical analysis (right panel). **(C)** Transwell assay showing migratory capacity of SiHa and HeLa cells after BAG2 overexpression (left panel), followed by statistical analysis (right panel). **(D)** Flow cytometry analysis of the effect of BAG2 overexpression on SiHa cells cycle progression. **(E)** Statistical plot of BAG2 knockdown and overexpression on G1 phase in SiHa cells. **(F)** Western blot analysis of proteins associated with cell cycle and EMT following BAG2 overexpression or knockdown in HeLa cells. **(G)** Overexpression efficiency of BAG2 lentivirus in U14 cell line detected by qRT-PCR. **(H)** Statistical plot of subcutaneous tumor volume in the control and BAG2 groups. **(I)** Representative image of popliteal lymph node metastasis model. Data are shown as mean ± SD, with n = 3 (B-C, E, G), 6 (A) or 8 (H) biological independent experiments. Statistical significance was determined by two-tailed unpaired Student's t-test (A-C, G-H). The one-way ANOVA with Dunnett's multiple comparisons test was utilized to ascertain statistical differences within the *BAG2* knockdown group, the two-tailed unpaired Student's t-test was used to assess the statistical difference for the BAG2 overexpression group (E).

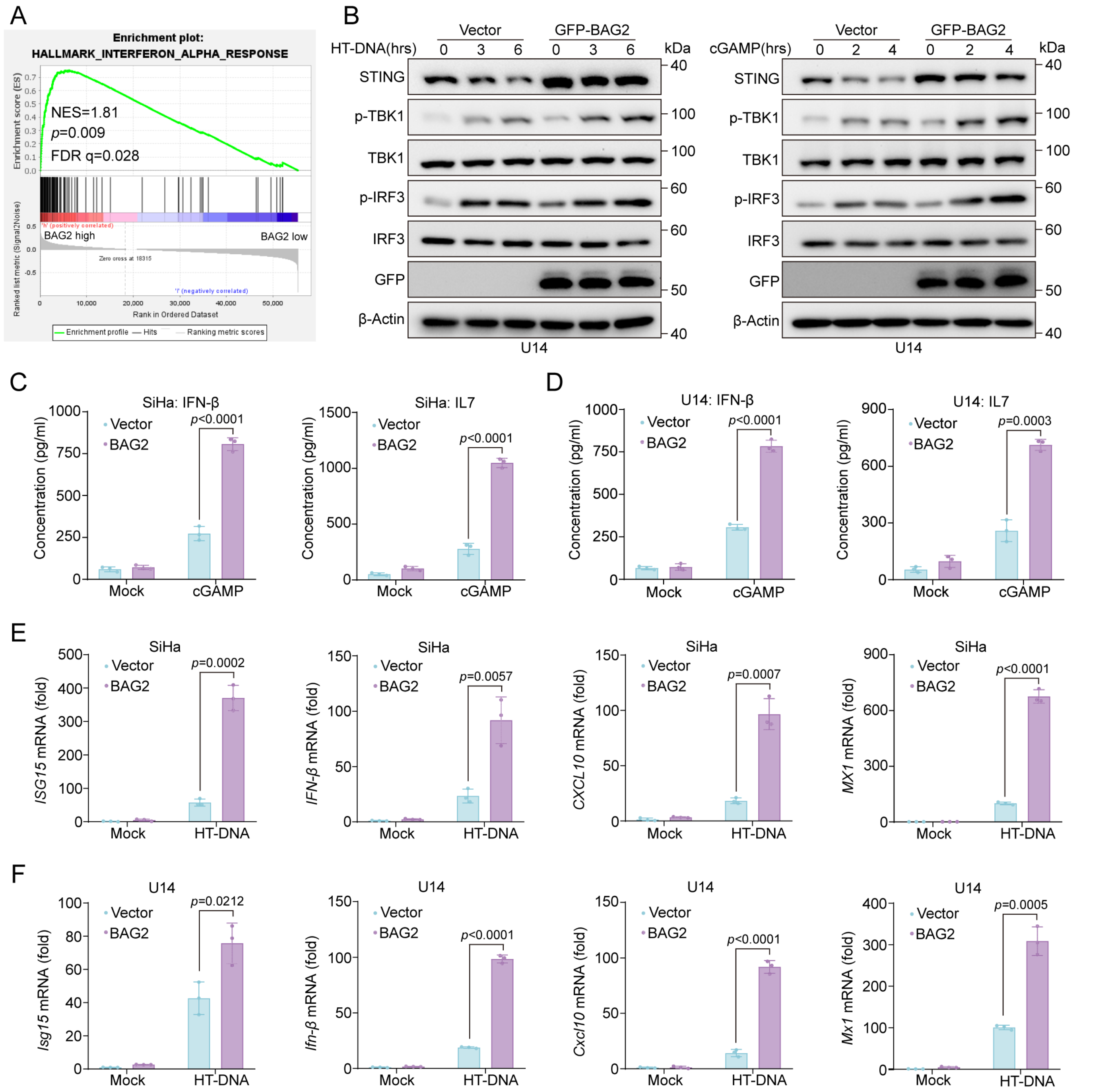

Figure S5. BAG2 is involved in STING-mediated type I interferon response.

(A) BAG2 related GSEA based on the TCGA database, and Hallmark gene sets were used as annotated gene sets. **(B)** U14 cells were transfected with vector or GFP-BAG2 for 48 hrs, and then they were treated with HT-DNA for 0, 3, 6 hrs or cGAMP for 0, 2, 4 hrs. Cells were collected for detection of STING, p-TBK1, TBK1, p-IRF3, and IRF3 protein levels by Western blot. **(C)** Vector or BAG2 was transfected in SiHa cells for 48 hrs, cell supernatants were collected by cGAMP treatment for 4 hrs, and IFN-β and IL7 production was measured by ELISA. **(D)** Vector or BAG2 was transfected in U14 cells for 48 hrs, cell supernatants were collected by cGAMP treatment for 4 hrs, and IFN-β and IL7 production was measured by ELISA. **(E-F)** SiHa cells and U14 cells were transfected with vector or BAG2 for 48 hrs, then HT-DNA was added for 6 hrs. qRT-PCR was used to measure the mRNA expression of *ISG15*, *IFN-β*, *MX1*, and *CXCL10*. Data are shown as mean ± SD, with n = 3 (C-F) biological independent experiments. Statistical significance was determined by two-tailed unpaired Student's t-test (C-F).

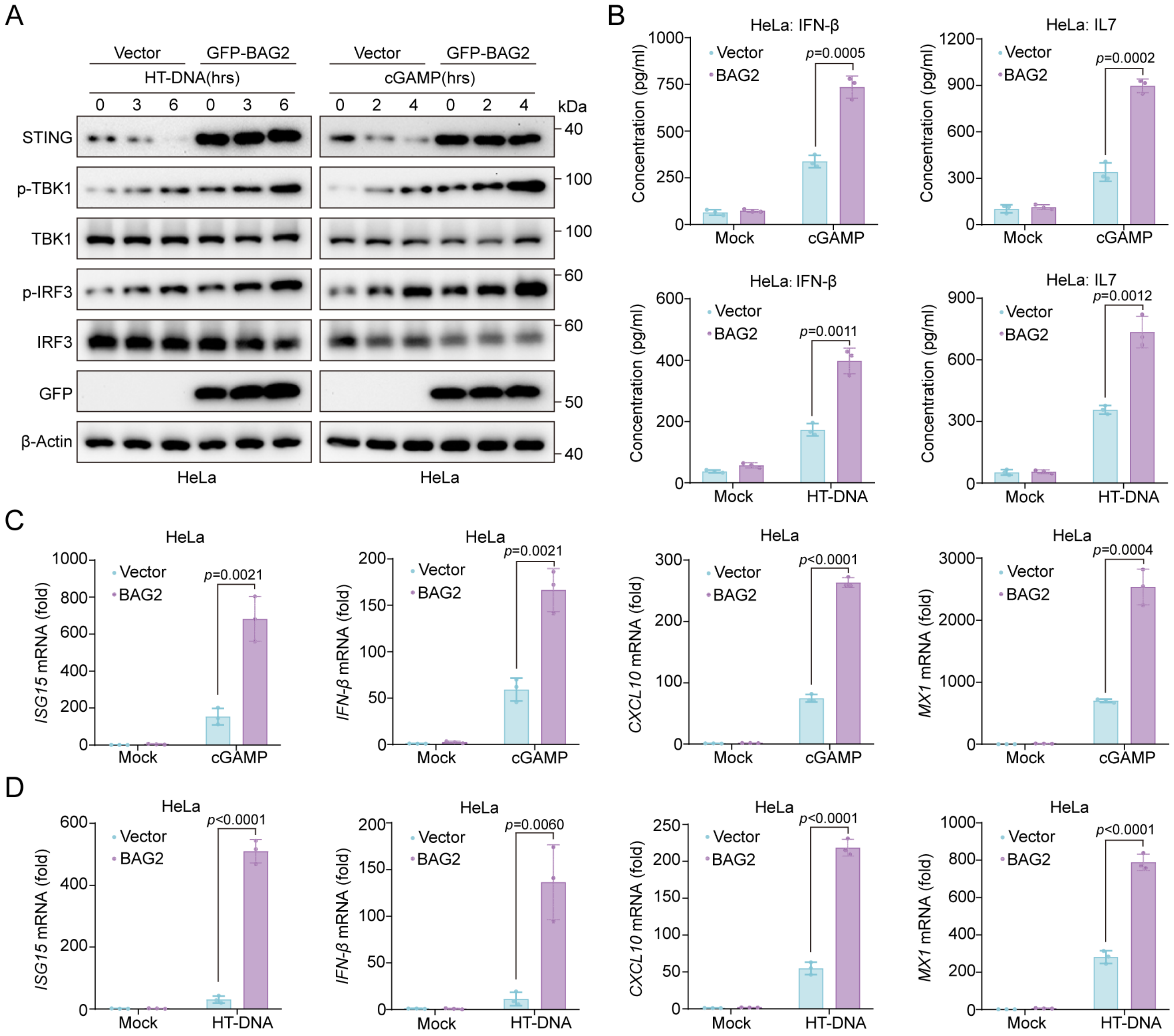

Figure S6. BAG2 is involved in STING-mediated type I interferon response in HeLa cells.
(A) HeLa cells were transfected with vector or GFP-BAG2 for 48 hrs, and then they were treated with HT-DNA for 0, 3, 6 hrs or cGAMP for 0, 2, 4 hrs. Cells were collected for detection of STING, p-TBK1, TBK1, p-IRF3, and IRF3 protein levels by Western blot. **(B)** Vector or BAG2 was transfected in HeLa cells for 48 hrs, cell supernatants were collected by cGAMP treatment for 4 hrs or HT-DNA treatment for 6 hrs, and IFN-β and IL7 production was measured by ELISA. **(C)** HeLa cells were transfected with Vector or BAG2 for 48 hrs, then cGAMP was added for 4 hrs. qRT-PCR was used to measure the mRNA expression of *ISG15*, *IFN-β*, *MX1*, and *CXCL10*. **(D)** HeLa cells were transfected with vector or BAG2 for 48 hrs, then HT-DNA was added for 6 hrs. qRT-PCR was used to measure the mRNA expression of *ISG15*, *IFN-β*, *MX1*, and *CXCL10*. Data are shown as mean ± SD, with n = 3 (B-D) biological independent experiments. Statistical significance was determined by two-tailed unpaired Student's t-test (B-D).

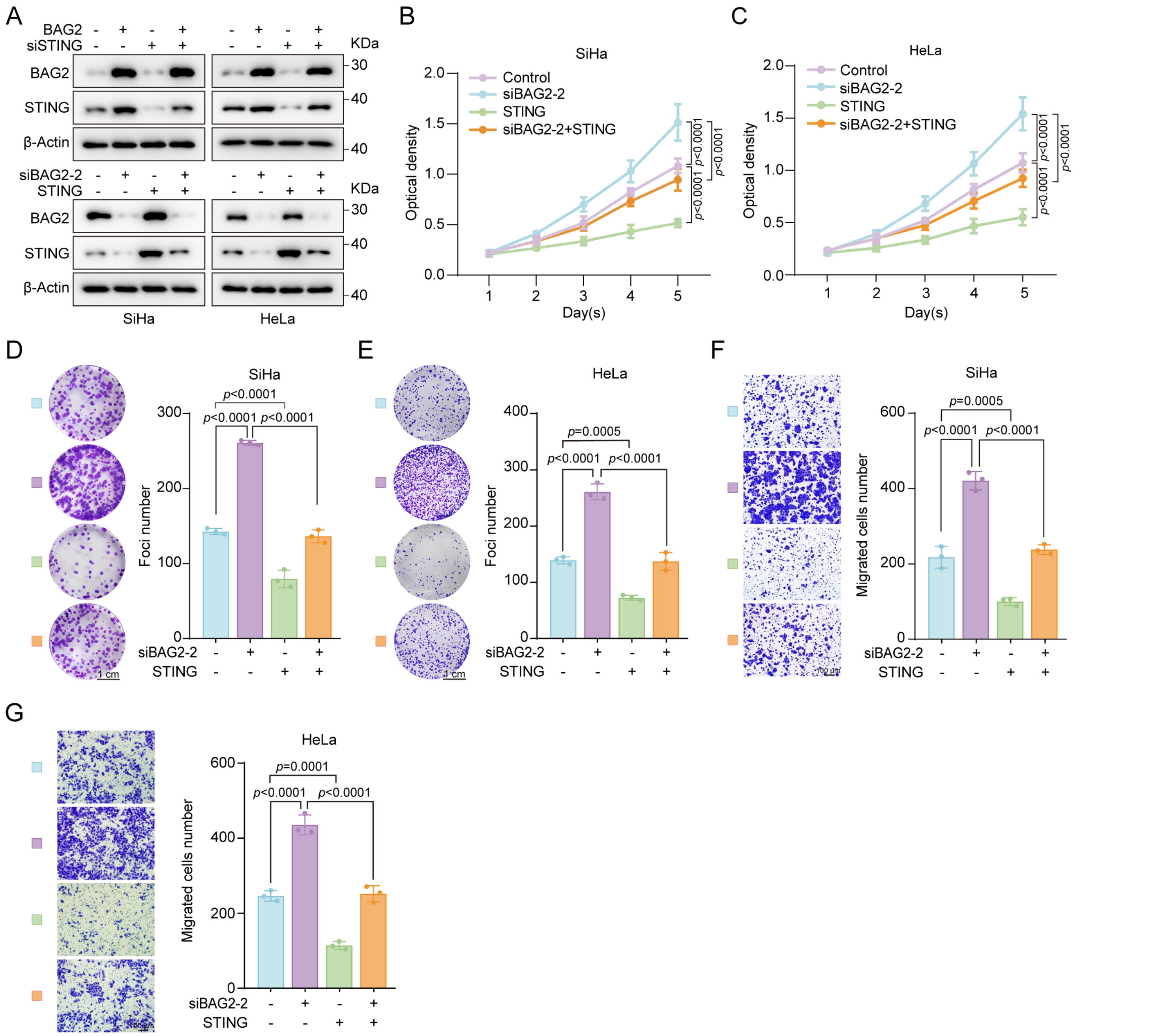

Figure S7. BAG2 inhibits cervical cancer proliferation and migration through STING.
(A) Protein levels of BAG2 and STING in each group were verified by Western blot in SiHa and HeLa cells. **(B-C)** The proliferation capacity of SiHa and HeLa cells after *BAG2* knockdown and/or STING overexpression was determined by CCK8 assay. **(D-E)** Foci formation assay of proliferative capacity of SiHa and HeLa cells after *BAG2* knockdown and/or STING overexpression treatment (left panel), followed by statistical analysis (right panel). **(F-G)** Transwell assay showing migratory capacity of SiHa and HeLa cells after *BAG2* knockdown and/or STING overexpression (left panel), followed by statistical analysis (right panel). Data are shown as mean ± SD, with n = 3 (D-G), 6 (B-C) biological independent experiments. Statistical significance was determined by one-way ANOVA with Tukey's multiple comparisons test (B-G).

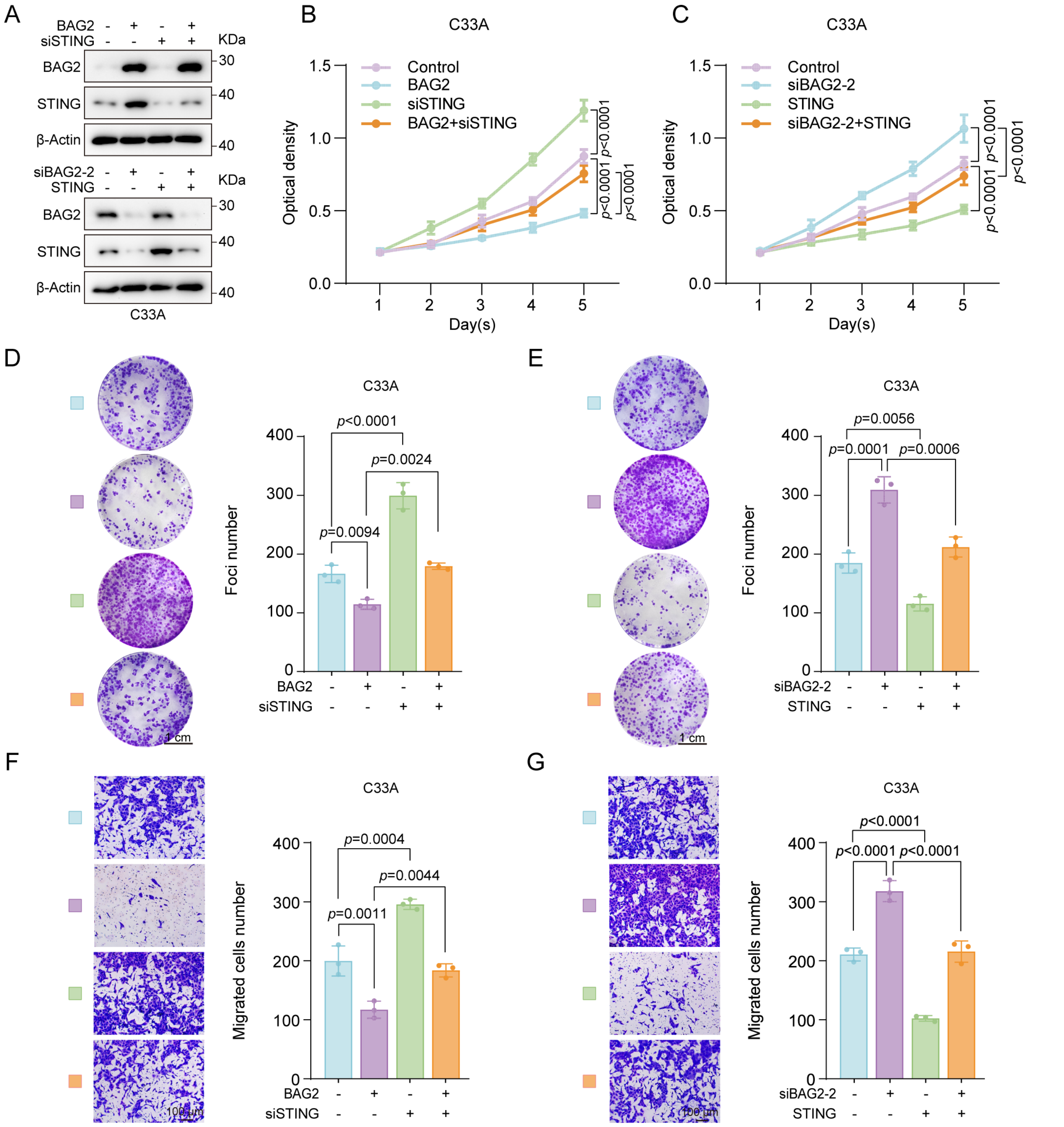

Figure S8. BAG2 inhibits cervical cancer proliferation and migration through STING in C33A cells. **(A)** Protein levels of BAG2 and STING in each group were verified by Western blot in C33A cells. **(B)** The proliferation capacity of C33A cells after BAG2 overexpression and/or *STING* knockdown was determined by CCK8 assay. **(C)** The proliferation capacity of C33A cells after *BAG2* knockdown and/or STING overexpression was determined by CCK8 assay. **(D)** Foci formation assay of proliferative capacity of C33A cells after BAG2 overexpression and/or *STING* knockdown treatment (left panel), followed by statistical analysis (right panel). **(E)** Foci formation assay of proliferative capacity of C33A cells after *BAG2* knockdown and/or STING overexpression treatment (left panel), followed by statistical analysis (right panel). **(F)** Transwell assay showing migratory capacity of C33A cells after BAG2 overexpression and/or *STING* knockdown (left panel), followed by statistical analysis (right panel). **(G)** Transwell assay showing migratory

capacity of C33A cells after *BAG2* knockdown and/or STING overexpression (left panel), followed by statistical analysis (right panel). Data are shown as mean ± SD, with n = 3 (D-G), 6 (B-C) biological independent experiments. Statistical significance was determined by one-way ANOVA with Tukey's multiple comparisons test (B-G).

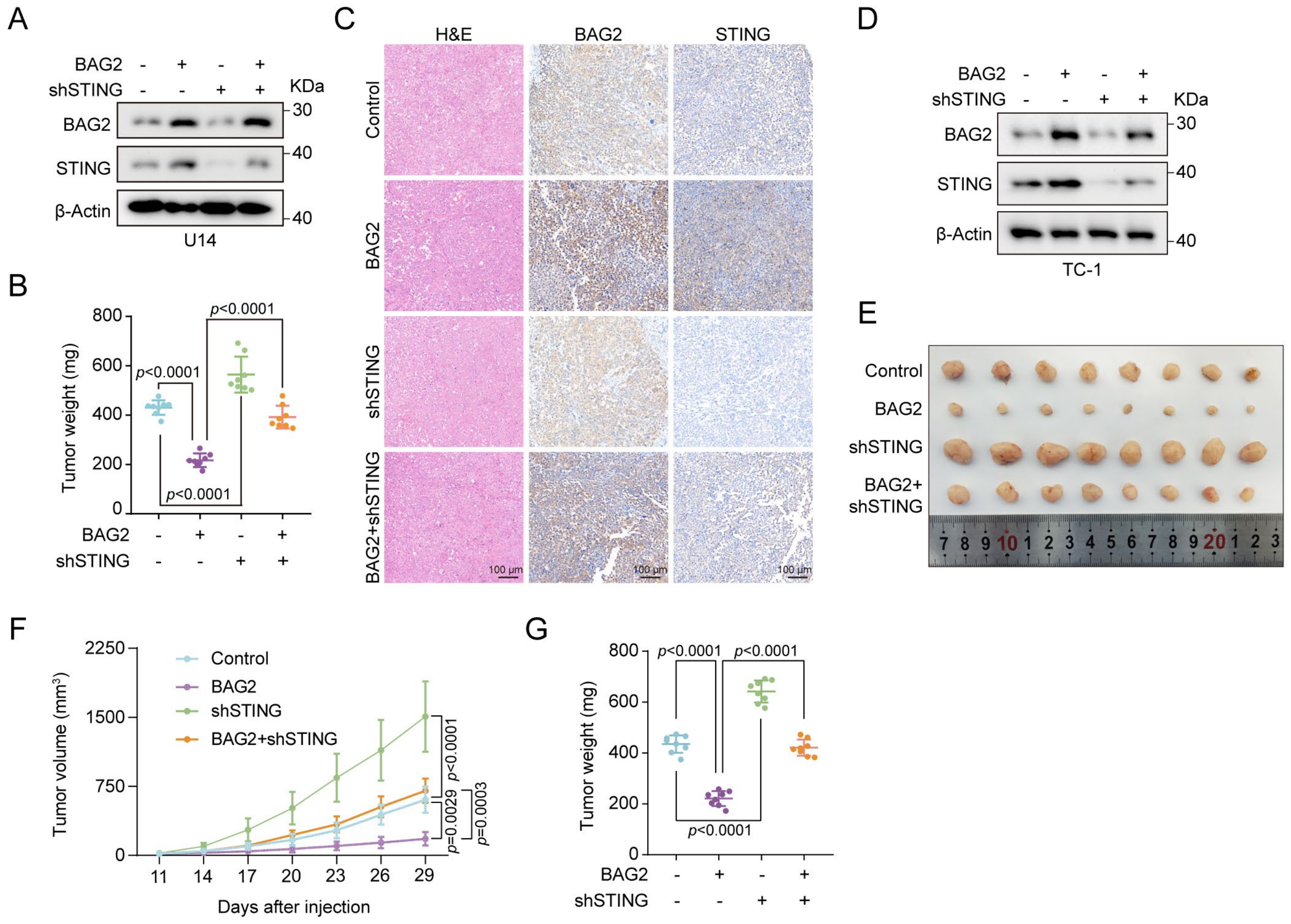

Figure S9. BAG2 inhibits cervical cancer cell proliferation through STING *in vivo*.

(A) Protein levels of BAG2 and STING in each group were verified by Western blot in U14 cells. **(B)** Weight statistics of subcutaneous tumors in U14 cells control, BAG2, *shSTING* and BAG2+*shSTING* groups. **(C)** Representative images of H&E and IHC staining analyses in subcutaneous tumor tissue of each group in U14 cells. **(D)** Protein levels of BAG2 and STING in each group were verified by Western blot in TC-1 cells. **(E)** Anatomical diagram of subcutaneous tumor tissue in different groups of TC-1 cells. **(F)** Statistical plot of subcutaneous tumor growth volume between groups in TC-1 cells. **(G)** Statistical plot of subcutaneous tumor weight between groups in TC-1 cells. Data are shown as mean ± SD, with n = 8 (B, F-G) biological independent experiments. Statistical significance was determined by one-way ANOVA with Tukey's multiple comparisons test (B, F-G).

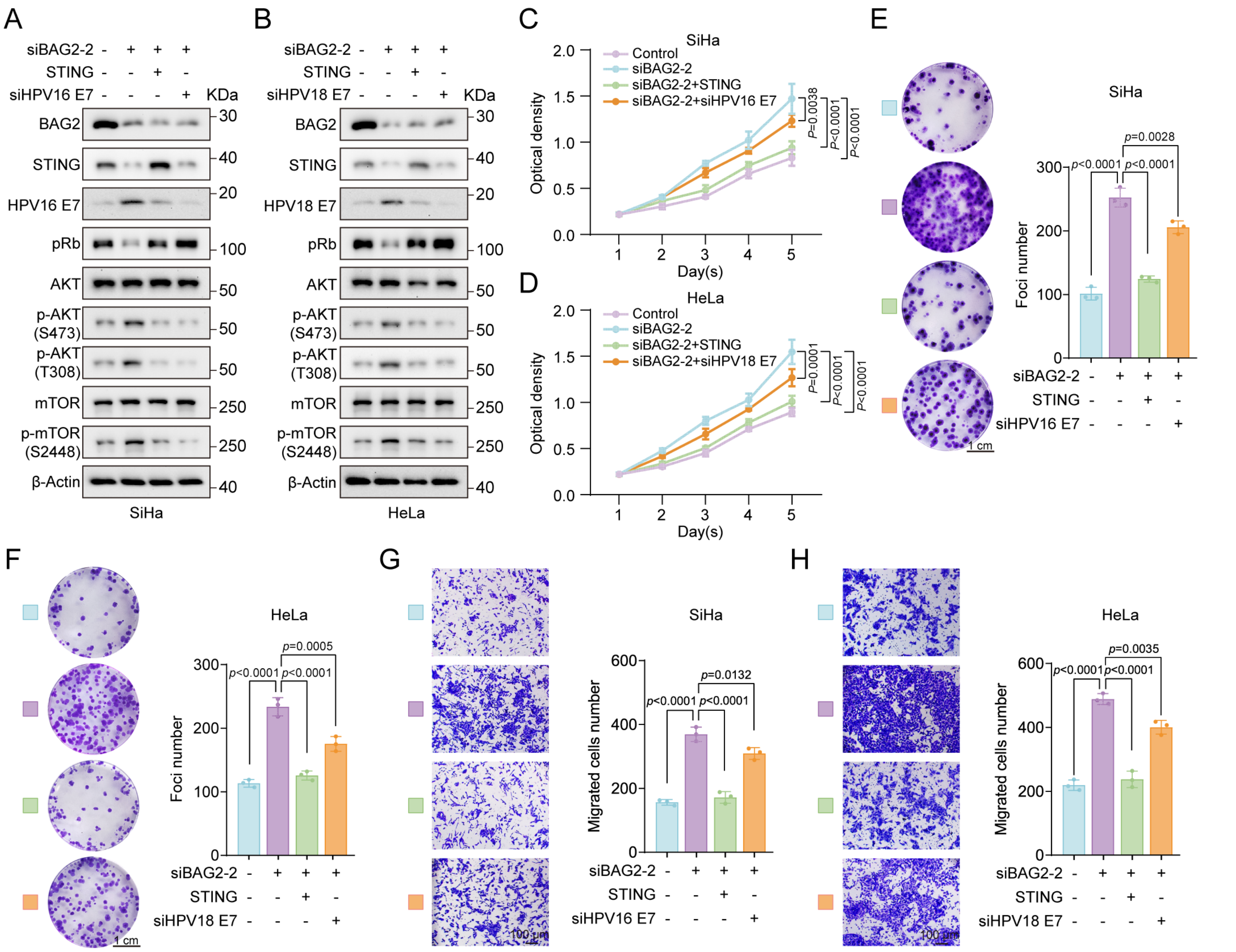

Figure S10. The tumor suppressor function of the BAG2-STING axis is only partially dependent on the STING-HPV E7 pathway.

(A) Western blot analysis of protein levels related to BAG2, STING, HPV16 E7, pRb and AKT signaling pathway in SiHa cells in control, *siBAG2*, *siBAG2*+STING and *siBAG2+siHPV16 E7* groups. **(B)** Western blot analysis of protein levels related to BAG2, STING, HPV18 E7, pRb and AKT signaling pathway in HeLa cells in control, *siBAG2*, *siBAG2*+STING and *siBAG2+siHPV18 E7* groups. **(C)** The proliferation capacity of SiHa cells in indicated groups was determined by CCK8 assay. **(D)** The proliferation capacity of HeLa cells in indicated groups was determined by CCK8 assay. **(E)** Foci formation assay of proliferative capacity of SiHa cells in indicated groups (left panel), followed by statistical analysis (right panel). **(F)** Foci formation assay of proliferative capacity of HeLa cells in indicated groups (left panel), followed by statistical analysis (right panel). **(G)** Transwell assay showing migratory capacity of SiHa cells in indicated groups (left panel), followed by statistical analysis (right panel). **(H)** Transwell assay showing migratory capacity of HeLa cells in indicated groups (left panel), followed by statistical analysis (right panel). Data are shown as mean ± SD, with n = 3 (E-H), 6 (C-D) biological independent experiments. Statistical significance was determined by one-way ANOVA with Tukey's multiple comparisons test (C-H).

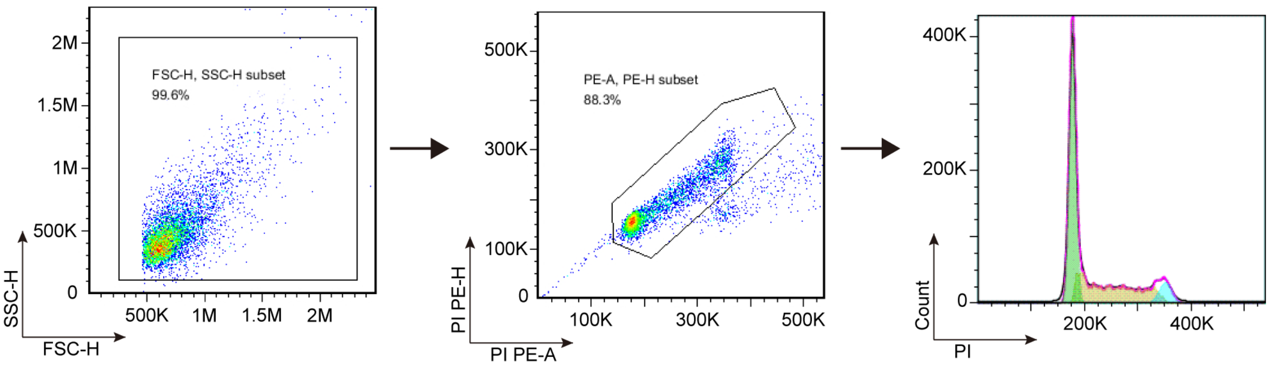

Figure S11. Gating strategy for flow analysis.
For the flow cell cycle analyses performed in this study, cell populations were identified using FSC and SSC (left panel), and then gates of this cell population were placed on PI PE-A and PI PE-H plots (middle panel), and, finally, cell cycle curves were fitted (right panel).

Table S1-S8

Table S1. Clinicopathological characteristics of immunotherapy cohort in Zhongnan Hospital.

Patient ID	Gender	Age / ys	Types of immunotherapy	Reactivity
1	Female	56	Sintilimab Injection	Sensitivity
2	Female	49	Camrelizumab Injection	Sensitivity
3	Female	58	Tislelizumab Injection	Sensitivity
4	Female	74	Sintilimab Injection	Sensitivity
5	Female	54	Camrelizumab Injection	Sensitivity
6	Female	54	Toripalimab Injection	Sensitivity
7	Female	44	Bevacizumab Injection	Resistance
8	Female	62	Sintilimab Injection	Resistance
9	Female	53	Toripalimab Injection	Resistance
10	Female	71	Camrelizumab Injection	Resistance
11	Female	55	Sintilimab Injection	Resistance
12	Female	69	Sintilimab Injection	Resistance
13	Female	56	Sintilimab Injection	Resistance
14	Female	53	Camrelizumab Injection	Resistance
15	Female	54	Bevacizumab Injection	Resistance
16	Female	43	Sintilimab Injection	Resistance
17	Female	50	Sintilimab Injection	Resistance
18	Female	86	Sintilimab Injection	Resistance
19	Female	59	Bevacizumab Injection	Resistance

Table S2. Detailed information on protein molecular docking interaction sites.

BAG2- STING interface residue pair(s)	Hydrogen Bonds(Å)
PHE 124-TRP 34	2.896
LEU 125-VAL 31	4.345
LEU 125-TRP 34	2.636
LEU 125-GLY 35	3.35
LEU 125-LEU 36	4.705
LEU 125-GLY 37	4.263
ASP 126-VAL 31	4.801
LEU 128-TRP 34	3.98
SER 184-TRP 34	3.348
ALA 187-LEU 44	4.918
ILE 188-LEU 30	3.21
ILE 188-TRP 34	2.928
LEU 190-PHE 105	4.515
LEU 191-LEU 44	2.631
LEU 191-LEU 47	2.53
GLU 192-LEU 30	3.063
SER 194-LEU 51	2.906
SER 194-LEU 101	4.481
LYS 195-LEU 47	4.091
LYS 195-LEU 51	3.944
ALA 197-GLN 55	3.428
ALA 197-LEU 98	2.417
GLY 198-LEU 51	4.825
GLY 198-LEU 54	3.682
GLY 198-GLN 55	2.927
GLY 198-LEU 58	3.93
GLY 198-LEU 98	4.454
SER 199-LEU 54	4.977
LYS 200-ARG 94	4.536
THR 201-GLN 55	2.507
THR 201-LEU 58	2.671
THR 201-ARG 95	3.194
THR 201-LEU 98	3.749
LEU 202-LEU 54	3.765
LEU 202-LEU 58	3.292
GLN 204-LEU 89	4.733
GLN 204-GLY 90	4.805
GLN 204-CYS 91	3.625
GLN 204-ARG 94	4.097
GLN 204-ARG 95	3.828
ASN 205-LEU 58	3.055
ASN 205-ASN 61	3.705
ASN 205-GLY 62	4.317
ASN 205-CYS 88	4.013

BAG2- STING interface residue pair(s)	Hydrogen Bonds(Å)
ASN 205-LEU 89	4.813
ASN 205-ARG 95	2.287
ALA 206-LEU 139	4.052
GLU 207-LYS 150	4.542
SER 208-ALA 87	3.422
SER 208-CYS 88	3.661
ARG 209-ASN 61	3.199
ARG 209-SER 65	3.616
ARG 209-LYS 137	3.246
PHE 210-LEU 139	3.093
PHE 210-ILE 144	3.512
PHE 210-VAL 147	3.524
ASN 211-ARG 76	3.494
ASN 211-PHE 153	3.173
ASN 211-GLU 286	4.006

BAG2-STING (Docking Score：-263.67, Confidence Score：0.9066, Ligand RMSD：74.81);
Amino acid pairs with bond lengths of 2.9A-3.1 Å were selected as the best binding sites.

Table S3. Clinicopathological characteristics of the Zhongnan Hospital cohort based on BAG2 protein level.

Patient ID	Gender	Age / ys	Tumor Size / cm^3	TNM Stage
1	Female	39	3 *1 *0.5cm	pT1b1N0Mx
2	Female	60	4.6*3*1cm	pT2bN1Mx
3	Female	74	4.2*3.5*3.0cm	T2aN0Mx
4	Female	70	4.5*3.5*1cm	pT2bN0Mx
5	Female	64	4*2.5*2cm	pT1b1N0Mx
6	Female	59	3*1.2*1.2cm	pT2aN0Mx
7	Female	65	3.4 *2 *1.4cm	pT1bN0Mx
8	Female	52	4.1 *3.7 *2.2cm	pT2bN1Mx
9	Female	50	3 *1.8 *1.1cm	pT1b1N0Mx
10	Female	56	4.1*3.7*2.2cm	pT1b2N0Mx
11	Female	54	3*2.5*1.7cm	pT1bN0Mx
12	Female	69	5 *4 *3cm	pT1b2N0Mx。
13	Female	57	2.2*1.7*0.5cm	pT2a1N0Mx
14	Female	61	3.5*2.5*2cm	pT2a1N0Mx
15	Female	50	5*3.5*3.5cm	pT2N0Mx

Table S4. Clinicopathological characteristics of the Zhongnan Hospital cohort based on *BAG2* mRNA level.

Patient ID	Gender	Age / ys	Tumor Size / cm^3	TNM Stage
1	Female	47	2*1.5*1.5	pT1bN0Mx
2	Female	55	4*3.5*3	pT1b1N1Mx
3	Female	54	5*4*1.8	pT2bN1M
4	Female	47	4.5 *4.5 *1.5	pT2aN1Mx
5	Female	41	6*3.2*1.5	pT1b2N0Mx
6	Female	70	3 *2.5 *1	pT1b1N0Mx
7	Female	59	3.0 *1.5 *0.7	pT1N1Mx
8	Female	54	2.5*2*2	pT1b1N0Mx
9	Female	52	3.3*3*2	pT2N1Mx
10	Female	74	4.3 *3.1 *2.5	pT1b2N0Mx
11	Female	49	4.5 *2.8 *2.2	pT1b2N0Mx
12	Female	76	3 *2 *1.5	pT2a1N0Mx
13	Female	70	2.9*2.5*0.5	pT1b1N0Mx
14	Female	66	2.2*1.1*0.6	pT1N0Mx
15	Female	66	4.2*2.8*1.5	pT2a2N0Mx
16	Female	44	1.8*0.8*0.7	pT1b1N0Mx
17	Female	65	3*2.5*1	pT2a1N0Mx
18	Female	54	2.2 *2 *1.8	pT2a1N0Mx

Table S5. Clinicopathological characteristics of the HUteS168Su01 cohort on BAG2.

	Variables	Total (n=119)	BAG2 high expression (n=60)	BAG2 low expression (n=59)	*p*-value	Statistics method (two-tailed)
Age (year) (%)	<=50	83 (69.75)	40 (66.67)	43 (72.88)	0.4606	Chi-square
	>50	36 (30.25)	20 (33.33)	16 (27.12)		
Grade (%)	<Grade III	27 (22.69)	15 (25.00)	12 (20.34)	0.2972	Chi-square
	Grade III	73 (61.34)	32 (53.33)	41 (69.49)		
AJCC stage (%)	<Stage II	95 (79.83)	53 (88.33)	42 (71.19)	**0.0198**	Chi-square
	>=Stage II	24 (20.17)	7 (11.67)	17 (28.87)		
N (%)	N=1	23 (19.33)	6 (10.00)	17 (28.81)	**0.0094**	Chi-square
	N0	96 (80.67)	54 (90.00)	42 (71.19)		
Recurrence (%)	No	82 (68.91)	46 (76.67)	36 (61.02)	0.0652	Chi-square
	Yes	37 (31.09)	14 (23.33)	23 (38.98)		

BAG2 expression group: The median of the BAG2 average optical density was cut-off value;

Statistical significance was determined by two-tailed Chi-square or two-tailed Fisher's exact test. No adjustments were made for multiple comparisons.

Table S6. Details of the antibodies used in this study.

Antibody	Catalog No.	Source	Dilution or amount
Flag-tag	F1804	Sigma	IP/1 μg WB/1:1000 IF/1:200
HA-tag	TA180128	Origene	IP/1 μg WB/1:1000
HA-tag	AE105	ABclonal	IF/1:200 WB/1:10000
GFP-tag	SC-9996	Santa Cruz	IP/1 μg WB/1:1000
GFP-tag	50430-2-AP	Proteintech	IF/1:200 WB/1:1000
Myc-tag	AE010	ABclonal	WB/1:1000
His-tag	10001-0-AP	Proteintech	WB/1:1000
GST-tag	10000-0-AP	Proteintech	WB/1:1000
BAG2	A8775	ABclonal	WB/1:1000 IHC/1:200
BAG2	ab79406	Abcam	IP/1 μg
STING	A21051	ABclonal	IP/1 μg WB/1:2000 IHC/1:200
STUB1	A11751	ABclonal	IP/1 μg WB/1:1000
Ubiquitin	ab7254	Abcam	WB/1:1000
β-Actin	66009-1-Ig	Proteintech	WB/1:20000
N-cadherin	13116	Cell Signaling Technology	WB/1:500
E-cadherin	3195	Cell Signaling Technology	WB/1:500
β-catenin	8480	Cell Signaling Technology	WB/1:1000
Vimentin	5741	Cell Signaling Technology	WB/1:1000
P27	25614-1-AP	Proteintech	WB/1:1000
CDK1	19532-1-AP	Proteintech	WB/1:2000
CDK4	ab108357	Abcam	WB/1:1000

Antibody	Catalog No.	Source	Dilution or amount
Snail	3879	Cell Signaling Technology	WB/1:1000
TBK1	A3458	ABclonal	WB/1:1000
p-TBK1	AP1418	ABclonal	WB/1:500
IRF3	A11118	ABclonal	WB/1:1000
p-IRF3	AP0623	ABclonal	WB/1:1000
AKT	4691	Cell Signaling Technology	WB/1:1000
p-AKT (T308)	9275	Cell Signaling Technology	WB/1:1000
p-AKT (S473)	4060	Cell Signaling Technology	WB/1:1000 IHC/1:200
mTOR	ab32028	Abcam	WB/1:1000
p-mTOR (S2448)	ab109268	Abcam	WB/1:1000
pRb	10048-2-Ig	Proteintech	WB/1:5000
HPV16 E7	sc-6981	Santa Cruz Biotechnology	WB/1:500
HPV18 E7	sc-365035	Santa Cruz Biotechnology	WB/1:1000
CD8	A11856	ABclonal	IHC/1:100
Ki67	A20018	ABclonal	IHC/1:200

IP: immunoprecipitation; WB: Western blot; IF: Immunofluorescence; IHC: Immunohistochemistry.

Table S7. Details of compounds used in the study.

Reagents	Catalog No.	Source
MG132	HY-13259	MedChemExpress
CHX	HY-12320	MedChemExpress
CQ	HY-17589A	MedChemExpress
CCK-8	A311-01	Vazyme Biotech
HT-DNA	D6898	Sigma
cGAMP	HY-12512	MedChemExpress

Table S8. Primers used for qRT-PCR.

Gene	Forward primer (5'-3')	Reverse primer (5'-3')
human BAG2	ATCAACGCTAAAGCCAACGAG	CGTCACTGATCTGCCTCATGT
human STING	CCAGAGCACACTCTCCGGTA	CGCATTTGGGAGGGAGTAGTA
human STUB1	AGCAGGGCAATCGTCTGTTC	CAAGGCCCGGTTGGTGTAATA
human CXCL10	GTGGCATTCAAGGAGTACCTC	TGATGGCCTTCGATTCTGGATT
human ISG15	CGCAGATCACCCAGAAGATCG	TTCGTCGCATTTGTCCACCA
human IFN-β	ATGACCAACAAGTGTCTCCTCC	GGAATCCAAGCAAGTTGTAGCTC
human MX1	GGTGGTCCCCAGTAATGTGG	CGTCAAGATTCCGATGGTCCT
mouse *Bag2*	AGACGCAGCTACTGCTGTTG	CGGATCGTTTCCACCGAGAC
mouse *Cxcl10*	CCAAGTGCTGCCGTCATTTTC	GGCTCGCAGGGATGATTTCAA
mouse *Isg15*	GGTGTCCGTGACTAACTCCAT	CTGTACCACTAGCATCACTGTG
mouse *Ifn-β*	AGCTCCAAGAAAGGACGAACA	GCCCTGTAGGTGAGGTTGAT
mouse *Mx1*	GACCATAGGGGTCTTGACCAA	AGACTTGCTCTTTCTGAAAAGCC
human/mouse β-Actin	ACACTGTGCCCATCTACGAG	TCAACGTCACACTTCATGATG
